# A systematic review of economic evaluations of interventions to tackle cardiovascular disease in low- and middle-income countries

**DOI:** 10.1186/1471-2458-12-2

**Published:** 2012-01-03

**Authors:** Marc Suhrcke, Till A Boluarte, Louis Niessen

**Affiliations:** 1 Norwich School of Medicine, University of East Anglia, Norwich NR4 7TJ, UK; 2 UKCRC Centre for Diet and Activity Research (CEDAR), Robinson Way, Cambridge CB2 0SR, UK; 3 Department of Medicine, University of Witten/Herdecke, Faculty of Health, Alfred-Herrhausen-Str. 50, 58448 Witten, Germany; 4 Department of International Health, Johns Hopkins Bloomberg School of Public Health, 615 N Wolfe Street, Baltimore, MD, USA

## Abstract

**Background:**

Low-and middle-income countries are facing both a mounting burden of cardiovascular disease (CVD) as well as severe resource constraints that keep them from emulating some of the extensive strategies pursued in high-income countries. There is thus an urgency to identify and implement those interventions that help reap the biggest reductions of the CVD burden, given low resource levels. What are the interventions to combat CVDs that represent good "value for money" in low-and middle-income countries? This study reviews the evidence-base on economic evaluations of interventions located in those countries.

**Methods:**

We conducted a systematic literature review of journal articles published until 2009, based on a comprehensive key-word based search in generic and specialized electronic databases, accompanied by manual searches of expert databases. The search strategy consisted of freetext and MeSH terms related to economic evaluation and cardiovascular disease. Two independent reviewers verified fulfillment of inclusion criteria and extracted study characteristics.

**Results:**

Thirty-three studies met the selection criteria. We find a growing research interest, in particular in most recent years, if from a very low baseline. Most interventions fall under the category primary prevention, as opposed to case management or secondary prevention. Across the spectrum of interventions, pharmaceutical strategies have been the predominant focus, and, taken at face value, these show significant positive economic evidence, specifically when compared to the counterfactual of no interventions. Only a few studies consider non-clinical interventions, at population level. Almost half of the studies have modelled the intervention effectiveness based on existing risk-factor information and effectiveness evidence from high-income countries.

**Conclusion:**

The cost-effectiveness evidence on CVD interventions in developing countries is growing, but remains scarce, and is biased towards pharmaceutical interventions. While the burden of cardiovascular disease is growing in these countries, future research should put greater emphasis on non-clinical interventions than has hitherto been the case. Significant differences in outcome measures and methodologies prohibit a direct ranking of the interventions by their degree of cost-effectiveness. Considerable caution should be exercised when transferring effectiveness estimates from developed countries for the purpose of modelling cost-effectiveness in developing countries. New local CVD risk factor and intervention follow-up studies are needed. Some pharmaceutical strategies appear cost-effective while clarifications are needed on the diagnostic approach in single high-risk factor vs. absolute risk targeting, the role of patient compliance, and the potential public health consequences of large-scale medicalization.

## Background

Cardiovascular diseases (CVD) make up the largest share of mortality and burden of disease across developed as well as developing countries, accounting for 30% of deaths and about 14% of DALYS lost [1]. In particular in developing countries the CVD burden is growing. Between 1990 and 2020, coronary heart disease alone is anticipated to increase by 120% for women and by 137% for men in developing countries [2]. This epidemiological transition cannot solely be explained by a rise in life expectancy or the tackling of other conditions, such as communicable diseases, but can also be attributed to an increase in risk factor prevalence in developing countries (in particular in urban regions), including smoking, risk-increasing dietary patterns and physical inactivity [3]. These harmful behaviors contribute to chronic conditions, such as hypertension, dyslipidemia and diabetes mellitus, which in turn act as risk factors for cardiovascular disease. The undoubtedly mounting burden raises the question, what if anything could be done about it.

While a number of evidence-based strategies have been applied in developed countries that have helped reduce the overall burden, it is an open question whether the same set of interventions and policies is applicable in a developing country context. The very limited resources available to health systems in developing countries, coupled with a range of health challenges that extend well beyond CVDs, are obvious key constraints to a major expansion of services. Taken together, the average level of expenditures on health care per person in low and middle income countries only amount to 2.4% of the budget per person in high income countries [4]. Hence the urgency to think particularly hard about how to maximize the health gains of any dollar invested in health care in those heavily resource constrained countries. In this article we review the evidence on value for money of interventions that try to address CVD. Importantly, we only include those studies in our review that have an explicit focus on one or more low and middle income country [5].

This is not the first attempt to capture this body of evidence. We build on major recent efforts to determine and develop cost-effectiveness estimates, most notably the 2006 *Disease Control Priorities Project (DCP2) *and the WHO initiative on *Choosing Interventions that are cost-effective (CHOICE)*, which both scrutinize potentially efficient strategies to tackle different chronic diseases and their risk factors in developing regions. To the best of our knowledge though, there is no recent study that has looked at this topic in a systematic way. Mulligan et al. [6] have provided a review of economic evaluations of interventions to address non-communicable diseases in developing countries, concluding that the evidence base was severely limited and what did exist was of low quality. The period covered by their review ended, however, in January 2003 and there is thus a case for updating the review, here with a focus on CVD, in the hope that an arguably growing attention to NCDs in developing countries has also led to more cost-effectiveness research on the topic. To anticipate the basic thrust of our findings: while there has certainly been a growing research interest in economic evaluations in developing countries supporting the investment in certain drug-based strategies to tackle (part of) the problem, major research gaps do remain, both in some geographical regions and in particular in the major domain of non-clinical interventions.

The focus of this study is to conduct a systematic review on the cost-effectiveness of interventions to address cardiovascular disease in developing countries, both at the population and individual level. We analyze the characteristics of the evidence, describe and discuss methods used in the research, and highlight other trends observed in the literature. In addition to describing what is known on the basis of the reviewed evidence we also seek to point out the deficits and gaps in the existing research. In particular we discuss the challenge of evidence transferability from developed to developing countries.

## Methods

### Literature search and study selection

We conducted a disease- and country-specific review, following the methodology of other standard systematic reviews in this area [5,7] and respecting the PRISMA statement [8] (Additional file 1). We searched the databases PubMed, EconLit, Embase, and NHS EED for relevant articles. The searches were conducted in August 2010. In addition, the references of retrieved articles were manually searched for further material. The relevant publications of the *DCP2 *project and *WHO-CHOICE *program were completely hand-searched for relevant articles. As the use of technical terms for indexing international literature in databases is often inconsistent or errant we defined a search strategy with high sensitivity but low specificity. The search strategy consisted of freetext and MeSH terms related to economic evaluation and cardiovascular disease. The resulting hits were filtered for the occurrence of the term "developing countries" or any country name defined as middle- or low-income country according to the World Bank definition. The search included all years up to 2009. The search strings are provided in the additional material (Additional file 2--Search strings applied for the review).

Studies were included in the review when they fulfilled all of the following inclusion criteria:

• Published during or prior to the year 2009

• Full economic evaluation, i.e., comparative analysis of costs and outcomes of at least two alternatives;

• Applied study (trial generating primary data or modeling of secondary data); reviews, letters, abstracts, methodological and general articles were excluded;

• Intervention targeting CVD (CVD endpoint or risk factor end point)

• Assessment of or application to the health care system of a developing country as defined by the World Bank;

• Journal articles, i.e., exclusion of books, HTA reports, grey literature;

• Published in English or any language feasible to be translated by the authors (i.e. German, French, Spanish, Italian)

We limited our analysis to evaluations published in journals to assess only those publications that have at least undergone some basic quality control.

### Data extraction and critical appraisal

We developed a checklist to extract data from the full texts. We collected details on the study design, type of economic evaluation, intervention target, type of intervention (primary prevention, secondary prevention, and case management), details of interventions, benefit measure, sponsorship, economic perspective, details on the target group, results of the intervention, and the comparator used. Quality assessment was based on the author's statements on applied methodology. The data extraction form did not include explicit quality ratings, i.e. in-depth evaluation whether stated methodology, e.g. applying societal perspective, was correctly performed. Since the transferability of results from one region to another (i.e. from developed to developing countries) was of particular interest to us, we analyzed in detail the origin of secondary data used in economic evaluations that apply models to generate results (epidemiological data, effectiveness data, data on resource use, data on costs). Each paper was independently read by one of two trained researchers. The researcher decided about final inclusion of the article. A random sample of 10% of articles was read by both reviewers to determine the inerrater reliability. The interrater reliability for inclusion was calculated using Kappa statistics (K). All articles included were read by the reviewers for extraction of certain data. After critical appraisal, information collected in data extraction forms were transferred to an electronic database.

### Data analysis

We analyzed the distribution of extracted categories of economic evaluations. All analyses were performed using SPSS 16.0 software (SPSS Inc., Chicago IL).

## Results

**Figure 1:**
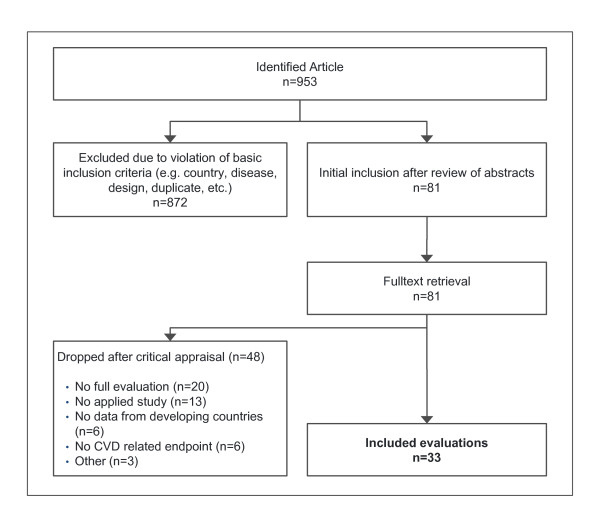
**Overview of in- and exclusion of studies**.

The systematic literature search initially identified 953 candidate articles, of which 81 were selected for fulltext retrieval (Figure 1 Overview of in- and exclusion of studies). The majority of articles were discarded at this initial stage mainly because they were duplicates, or it was obvious from the bibliographic data that they violated basic inclusion criteria. Fourty-eight articles were dropped after the critical appraisal because they failed one or more inclusion criteria. Finally, 33 articles were included in the review, of which basic results were extracted to a table in the additional material (Additional file 3--Descriptive table of results). Interrater reliability (K) was assessed on a sample of 10% of studies (n = 9). K was 0.78 for study inclusion, i.e., fulfillment of all entry criteria, indicating a very good level of agreement [9].

### Basic characteristics of included studies

The number of studies conducted has increased sharply in most recent years, if from a very low baseline. Most of the studies included in our review were published within the last 4 years of the review period. While only 13 articles had been published until 2005, there were 20 economic evaluations published from 2006 to 2009. A majority of 82% (n = 27) of the studies referred to a single country, compared to 18% (n = 6) of multi-country evaluations. Most of the single nation studies were conducted in South Africa (n = 7) and Brazil (n = 7), followed by Thailand (n = 3). Studies evaluating a multi-national setting, most often used an aggregated perspective at the level of WHO or World Bank regions (n = 4), while two evaluations evaluated strategies in health care settings based in different countries.

**Figure 2:**
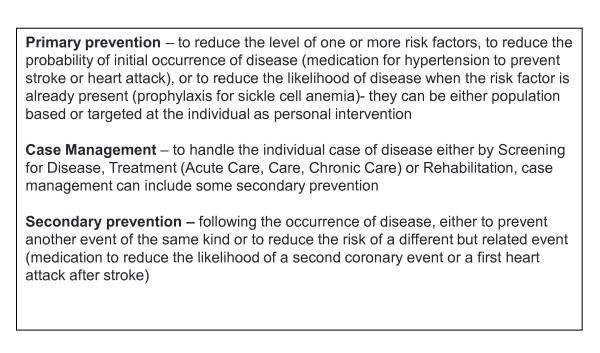
**Definition of types of intervention**.

Primary prevention was the most frequent type of intervention (n = 11), followed by case management of disease (n = 8) and secondary prevention (n = 5). Secondary prevention in our study is defined according to the guidelines of the American Heart Association [10], i.e. as a preventive intervention after an established heart disease has been diagnosed (e.g. coronary artery disease) (Figure 2). Nine studies scrutinized various types of strategies to combat cardiovascular disease. All but one case management strategy concerned treatment (n = 7), one study focused on rehabilitation (n = 1), while no screening strategy had been evaluated. Studies in the domain of primary prevention evaluated most often personal interventions (n = 6) and less often population-based interventions (n = 3), while two studies evaluated an intervention based on both approaches.

A closer look at the detailed characteristics of the interventions reveals that most measures involved pharmaceutical strategies (n = 14), followed by articles evaluating different procedures for the treatment of CVD (n = 7), e.g. stent implantation vs. by-pass-graft surgery. Few studies considered personal health education (n = 3) or population-based social marketing (n = 2). Four studies evaluated multiple types of interventions, such as pharmaceutical interventions, legislation and social marketing combined.

Clustering the studies by intervention targets, the vast majority of studies (n = 24) aimed at the reduction of risk factors. Among those, a combination of multiple risk factors has most often been addressed (n = 11), followed by interventions targeted at high blood pressure (n = 9). Other important risk factors, such as smoking (n = 1), physical inactivity (n = 1), and dyslipidemia (n = 1), were rarely evaluated. Other intervention targets were revascularization (n = 6) or fatal arrhythmia (n = 1).

### Characteristics of economic methods

Looking at the methods of economic evaluations, we observed that the authors most often used cost-effectiveness analysis with a clinical outcome (CEA--Clinical), such as a biomarker or change in health behavior as benefit measure (30%, n = 10). Cost-consequence analysis (CCA) (n = 10), has been an equally frequently used method. CCAs usually list benefits and costs of an intervention, without presenting them as a ratio, making it more complicated to compare different alternatives. Only about a third of studies used a more comprehensive approach to scrutinize interventions: those included eight cost-utility analyses (CUA) and four cost-effectiveness analyses with "life years gained" (CEA--Life years) as the benefit measure. The method of cost-benefit analysis (CBA) has not been applied at all.

In about half of the articles published, modeling was used to generate costs and benefits (n = 18). By contrast, seven studies conducted their evaluation alongside observational trials and four other evaluations generated their data alongside randomized controlled trials (RCT). Four studies used original data generated alongside other types of studies--mostly cross-sectional studies.

The perspective of an evaluation is important to the decision maker, in order to determine to whom the costs occur. This matters because an intervention might be cost-effective from one point of view (e.g. a societal one), but not from another (e.g. a health care provider view). Clearly stating the perspective adopted is therefore an essential task for the researcher(s) and is consistently recommended in guidelines of economic evaluations. In our review, 15 out of 33 studies did not follow this recommendation. Among those evaluations fulfilling this task, seven adopted the perspective of the health care provider and nine applied that of a third party payer (e.g. health insurance). Only two studies stated to have applied the patient's perspective, while no author explicitly adopted the most comprehensive view--the societal perspective.

### Funding of studies

Most studies (n = 19) did not mention their source of funding. Those who did, were supported either by the government (n = 5), the industry (n = 3), or by a foundation (n = 3). Three studies received financial support by other sources, such as universities or explicitly did not receive external funding.

### Use of secondary data

As mentioned above, 18 studies used modeling to evaluate interventions targeted at the CVD burden in certain developing countries. We conducted an in depth analysis of secondary data used in those models in order to determine the level of adaption to the country under analysis, as well as the reliability of data. Among those studies stating the source of epidemiological data in their analysis (n = 17), 14 used available data on disease burden and mortality from the country or region the analysis was located in and three articles used this type of data from developed countries.

A different picture emerges when looking at the data used for the calculation of intervention effectiveness. All 18 articles used information from studies mainly conducted in developed countries. While twelve studies based their model on the results of international systematic reviews, five studies relied on a single large randomized controlled trial (RCT) to estimate the effects of an intervention. One study used the results of an observational trial as evidence for effectiveness. In those studies that did state the origin of data for the calculation of "resource utilization" (n = 15), ten used data from the country the study was set in, compared to five evaluations that had incorporated data from developed countries into their model. All studies included in this sub-selection did use price data (for the goods and services used in the intervention) from the country or region the study was located in.

### Economic evidence for interventions

We explicitly refrained from ranking the interventions based on their cost-effectiveness ratio, in light of the diversity in methods and outcome measures applied across studies as well as the often considerable differences in methodological quality among studies (e.g. transparency on type of costs included). Nevertheless, we applied a simplified method--i.e. the so-called "hierarchical decision matrix" (Table 1)--that has been recommended to summarize results of systematic reviews of economic evaluations [11]. As a first step, outcomes on health benefits and costs against the comparator are extracted from the studies. Secondly, based on the findings, the evaluation results are allocated to 1 out of 9 categories, depending on their cost/health outcome profile. Each category defines how costs and health benefits of the intervention compare to its comparator, i.e. higher, equal, below. Considering all possible combinations, 9 categories are available for allocation--reaching from "higher costs/lower health benefits" to "less costs/higher health benefits". Finally, the matrix lists the different groups of intervention types/measures and counts the number of studies within the different cost/effectiveness categories. The categories are grouped so that they inform the decision maker on recommendations, i.e. "reject intervention", "neutral", "incremental analysis required", or "accept intervention". Details of results, including the reported cost-effectiveness ratios, are listed in the additional material (Additional file 4--Table of detailed results from included studies).

**Table 1:** Hierarchical Decision Matrix of interventions to tackle CVD in low- and middle income countries (by type of intervention and by type of intervention measure)

	Reject intervention			Incremental analysis required	Neutral	Incremental analysis required	Accept intervention		
**Health benefits**	**-**	**-**	**○**	**-**	**○**	**+**	**○**	**+**	**+**

**Costs**	**+**	**○**	**+**	**-**	**○**	**+**	**-**	**○**	**-**

***By type of Intervention***									

Case Management	0	0	0	1	0	5	2	0	0

Primary Prevention	0	0	0	0	0	9	1	0	1

Secondary Prevention	0	0	0	0	0	4	0	0	1

Various	0	0	0	0	0	7	2	0	0

***By type of Intervention Measure***									

Pharmaceutical	0	0	0	0	0	12	1	0	1

Procedure	0	0	0	1	0	3	2	0	0

Health Education (Personal Level)	0	0	0	0	0	2	0	0	1

Social Marketing (Pop. based)	0	0	0	0	0	2	0	0	0

Medical Technology	0	0	0	0	0	2	0	0	0

Health Care Delivery	0	0	0	0	0	0	2	0	0

Various	0	0	0	0	0	4	0	0	0

**+ **above comparator **○ **equal to comparator - below comparator

Numbers in cells are number of studies relevant to each permutation

According to our matrix, no study recommended the "rejection" of any intervention. Further, the vast majority of studies recommend "incremental analysis" to decide on the final acceptance of a measure, e.g. by defining an appropriate cost-effectiveness acceptance threshold.

Only one intervention measure, i.e. health care delivery, was uniformly recommended for "acceptance", followed by "procedures", with 40% of those studies recommending acceptance according to the matrix. This is not surprising, since these interventions targeted mostly at reducing costs while at least maintaining the quality of care.

Among all other types and measures of intervention, no clear recommendation could be established and incremental analysis is thus recommended for those cases.

## Discussion

### Content aspects

The observed distribution of studies over time supports the impression that the evaluation of cost-effective strategies to combat cardiovascular disease in developing countries has been a neglected topic for decades. However, around the time of the release of the second edition of the report on Disease Control Priorities (DCP2, April 2006), which among other issues covered interventions to address chronic diseases in developing countries, the number of publications on cost-effective strategies to reduce the burden caused by CVD in low- and middle income countries increased significantly. This could mean that the work on DCP2 has directly led to the publication of relevant work and/or that it has spurred the research interest in this area.

Nevertheless, large research gaps do remain in the area of economic evaluations. While certain countries in Latin America, Africa, Europe and South Asia have been subject to some formal assessment, there are regions in the world that have only been studied from an aggregate perspective. These countries are typically located in the regions of North Africa and the Middle East as well as Central Asia, South Asia and East Asia. This may reflect deficits in our research strategy (e.g. due to non-coverage of relevant languages), or--perhaps more likely--it may indeed mean a paucity of research efforts.

Ideally, a systematic review of this kind should provide an answer to the question: which are the most cost-effective ways of addressing CVD in developing countries? While we cannot provide a satisfactory answer to this question, simply because the evidence base is too limited, not enough transparent, and incomplete, we are in a position to describe for which strategies there is arguably strong evidence and where it is that research is missing.

There is significant evidence for pharmaceutical strategies to tackle risk factors as part of secondary prevention and--in some cases--also for primary prevention. While there appears to be a consensus on the utility (up to a point) of certain pharmaceutical strategies in general and the need for some form of their scaling up in developing countries [12], the debate continues to revolve around specific implementation and organisational issues [13]. This includes the discussion between those advocating the targeting of patients with a single but high risk factor (e.g. high blood pressure) on one hand and those arguing for an overall absolute risk approach (e.g. on the basis of 10-year risk of CVD), independent of the particular risk factor, on the other hand. There are also diverging views around the introduction of a 'poly-pill', a medication consisting of multiple pharmacological agents at a fixed dose, as a means to provide more generic treatment options (compared to treating each individual risk factor with a specific drug and dose). Some argue that the poly-pill would allow a broader population to access and use pharmacological care, due to lower requirements in risk factor assessment and monitoring. Even though large trials in developing countries have been undertaken to prove the effectiveness of the poly-pill approach [14], its overall consequences that would capture potential adverse effects, the impact on health inequalities, the consequences of mass medicalization for healthcare budgets in developing countries, with a lot of resources allocated to a few major medications, as well as the role of patient compliance, still awaits a thorough assessment.

By contrast, there has been remarkably little research coverage and discussion of non-clinical, population based approaches, e.g. health promotion through social marketing, or legislative actions as a way to tackle CVDs in developing countries.

Apart from the methodological issues in evaluating these interventions, at least two reasons may help explain this bias in the research. First, research on population based, non-clinical interventions is likely to be subject to a market failure: private actors do not have the incentive to engage in such research, because (a large share of) the resulting knowledge would become a public good that everybody could use, without having to pay the often substantial research costs to arrive at that knowledge. From a sheer economic efficiency (and not even from a moral or public health) perspective, this type of knowledge will be undersupplied compared to the social optimum [15]. An analysis of the funding sources of the articles included in our review shows that out of the three studies reporting industrial support, two evaluated pharmaceutical interventions and one a medical technology. (Given the few studies reporting a funding source, this observation may of course be of limited generalizability.)

Second, primary data on the effectiveness of specific population interventions typically does not exist for certain countries or regions. Since the results of interventions targeted to change defined health behaviours or implementing social marketing are highly dependent on cultural, infrastructural and other system-related aspects, scientists often argue that it is less feasible than in clinical evidence to transfer such results from developed regions to developing regions [16]. It is a widely held assumption in pharmaceutical research that a drug affecting biomedical processes would have approximately identical effects, irrespective of the ethnic context in which it is applied. We will scrutinize this hypothesis below, when considering the transferability of results among countries and regions.

Despite our general endorsement of some form of scaled up pharmaceutical support, it is also important to be mindful of the limitations of such a strategy. This is to do with the observation that any approach that defines the benchmark risk level (e.g. on blood pressure) as high as most current approaches recommend, inevitably misses out the typically large amount of people that is below that threshold but nevertheless shows ailments that are related to their (less than nominally "too high") risk factor levels (e.g. blood pressure) [17]. Even though an approach assessing the total absolute risk of individuals would address some of the issues, it would imply the allocation of (possibly disproportionately) large resources to healthcare for the elderly population due to the high contribution of age to these risk calculations. Clinically managed chronic care often is expensive and may be required for the remaining lifetime. An extension of the target group for treatment, though clinically justified, would cause higher pressures on the already constraint budgets of LIMCs. A population-based approach, such as reducing salt intake, would at least in principle also effect change in the entire population in the long term and not only in the highest risk group. Therefore including the larger group of beneficiaries in this outcome calculation might in some cases render such approaches attractive because it could be more cost-effective. This could be the case even when the overall population risk reduction is limited [18]. However, strong local level evidence of such a shift to proof these approaches cost-effective, accounting for obstacles in large-scale implementation and financing is still missing and requires further analysis.

In our search we found only a small number of studies assessing strategies to combat tobacco use. This is surprising in light of the otherwise well-established evidence on cost-effective strategies to address smoking-related health loss. In particular, taxation and legislation options have been rather well evaluated, certainly for developed regions and countries but also in developing countries [19]. The achieved reduction of smoking rates is shown to have lowered the burden of disease caused by CVD by about 36% in the UK [20]. One explanation for the few studies we identified might lie in our search strategy, which focused on studies concerning primarily CVD. Smoking interventions, by contrast, might be more often labeled in connection with lung diseases or as an independent disease. Indeed, upon closer scrutiny, more evidence for efficient strategies to reduce smoking in developing countries does exist. Those include other review articles, as for instance the 2003 study by Shibuya et al. [21] or the Chapter in the DCP2 publication by Jha P et al. [22], both of which describe an increase in tobacco tax as the most cost-effective strategy to reduce smoking prevalence, followed by comprehensive advertisement campaigns and bans on smoking in public places.

In addition, we were surprised to realize that contrary to an earlier review of ours on economic evaluations of primary prevention of CVD in developed countries [5], in the present review there were nearly no studies evaluating the effects of statins--alone or in combination with other drugs--on dyslipidemia. Neither for primary, nor for secondary prevention did our search reveal any such evidence for developing countries. Even though the first statin, Lovastatin, went off-patent in the US and Europe in 2001, it took until the patent expiration of the popular drug Simvastatin in 2006, for a statin (Simvastatin for high risk patients) to be added to the *World Health Organization Model List of Essential Medicines *in 2007 [23]. Until then, evaluations of strategies targeting dyslipidemia through statins might not have appeared useful for developing countries, since broad access to the drug had not been feasible. In addition, the diagnostic costs for determining blood lipid levels are relatively high, when compared for instance to measuring blood pressure. Therefore targeting specifically dyslipidemia is less feasible in developing countries, since the direct costs (and infrastructure costs) for diagnosis and monitoring of patients would require a considerable share of the scarce resources.

It may have come as a surprise that we found more studies in our review on primary prevention than on secondary prevention of CVD. In interpreting these results, however, it has to be acknowledged that we used the definition of secondary prevention as stated by the American Heart Association [10], i.e. meaning treating risk factors in patients with established cardiovascular disease (e.g. ischemic heart disease). Some other disciplines, e.g. public health, tend to follow a broader definition of secondary prevention that includes any treatment of evident hypertension or dyslipidemia [24]. Another influential institution, i.e. the European Medicines Agency [25], regards these definitions of prevention in CVD as artificial and outdated and prefers to discuss overall CVD risk on a continuum which needs to be tackled by suitable measures.

### Methodological aspects

In general, few of the studies adopted a comprehensive perspective in their analysis. The more comprehensive a study is, the easier it is for decision makers to compare the intervention to other alternatives available for funding. This applies to the computation of health benefits as well as to the economic perspective adopted. Only 12 out of the 33 studies included in our review used comprehensive units such as "life years gained" or the surrogate measure of "QALYs" or "DALYs" in their analysis. The remaining studies preferred biomarkers or CVD related incidents, which are easier to measure, but harder to compare to other interventions within or outside the health care sector. No article applied a cost-benefit approach.

Moreover, it is surprising to find that no study explicitly applied a societal perspective to the evaluation. Both the cost-benefit method and the societal perspective would in principle be helpful approaches for decision makers, in particular in developing countries. In these regions budgets are even more constraint and investments in healthcare compete heavily with those in other budgetary sectors, such as education or public infrastructure. To support the decision maker in her task of allocating resources across and within sectors, more comprehensive and hence more comparable evaluations might have been desirable.

Modeling is a useful or indeed often necessary method to produce economic evaluations in particular when certain data is missing or when long-term results represent a core interest of the analysis. Eighteen out of 33 studies included in our review used some form of modeling. In modeling, one main decision concerns what effect measure to apply to compute intervention outcomes. In general, in our review, both efficacy or effectiveness studies are used. Revicki and Frank analyzed the importance of both types of studies for pharmacoeconomic evaluations [26]. While efficacy studies or RCTs demonstrate the performance of an intervention under ideal and controlled conditions, effectiveness studies show the impact of treatments under regular clinical conditions or "real world" circumstances. Efficacy studies focus on the internal validity of results and therefore accuracy of conclusions--however, their practical use is limited due to potential lack in the generalisability of their results. Effectiveness studies have a more real-life set-up and lead to results of more practical value, increasing external validity--at the potential cost of internal validity. Revicki and Frank conclude that cost-effectiveness studies with RCTs "may provide a very precise answer to the wrong question" [26]. In general, efficacy rates are higher than effectiveness rates, therefore giving the decision maker a biased impression on success within his population of concern. Due to the lower adherence to treatment guidelines (by doctors and patients), co-morbidities and limited patient monitoring, efficacy rates usually drop in effectiveness trials [27,28]. Goldenberg and Glueck [29] reviewed retrospective studies concerning the goal attainment for statin therapy in managing CVD and found that ~20% of patients did not receive the necessary medication by their doctors when compared to guidelines. Furthermore, only 50% of treated patients achieved lipid-lowering goals with significant consequences for CVD mortality and morbidity. Another study focused on the patient side of adherence in an Italian population of 10,890 patients [30]. Only half of the patients who started on statins, continued to take the medication after 1 year. In patients for primary prevention of CVD, only 19% adhered to the regimen prescribed by the doctor. Predictors for non-adherence were younger age, total number of daily drug doses and having multiple prescribing physicians. Similar adherence rates can be found in the context of developing countries. Bowry et al. [31] systematically reviewed studies on the adherence to cardiovascular medication in resource-limited settings and found an average adherence rate of 58% according to pill-count and self-reporting. Common predictors of non-adherence to medication were poor knowledge, negative perceptions about the medication, occurrence of side-effects, high medication costs, and lack of family support. Factors such as age, gender, lifestyle, complex treatment regimens, and lack of access to health care services were not consistently associated with non-adherence.

Revicki and Frank conclude that RCTs are a precondition for conducting effectiveness studies. However, for providing the decision maker with relevant information about the pharmacoeconomic outcomes of an intervention, evaluations based on RCTs are of limited use, particular in a community setting [26].

In our sample of studies applying a modeling approach, 17 out of 18 studies incorporated large RCTs to calculate health benefit outcomes, either as single source or in metaanalyses. Moreover, these studies were all conducted in developed countries instead of the country under analysis or a country with similar conditions. This problem of transferring results will be discussed in detail below.

### Transferability of results between regions--opportunities and limitations

Conducting original economic evaluations for every intervention in every LMIC is well beyond the means of most developing countries' monetary and human resources. This general lack of capacity has to be considered when analyzing the studies and drawing conclusions. For example, a third of studies included in our review were conducted by authors at institutions in solely developed countries (n = 11).

Hence the idea of transferring results from one country to another, in particular from developed to developing countries, has always been a potentially attractive and fairly widely accepted alternative for researchers and decision-makers. However, this approach also bears several challenges, especially including differences in health system costs across countries, differential effectiveness of the same intervention, differential disease prevalence, differential valuation of outcomes, and differential efficiencies in the implementation of interventions. In what follows we discuss these issues in light of the existing cost-effectiveness evidence for CVD interventions.

#### Use of external data

Disease modeling is widely applied in research on developing countries, as is shown extensively in our review. Modeling approaches transfer data on disease epidemiology, risk factor associations, relative clinical efficacy, resource utilization, and unit cost from the country where the original study took place to a target country of interest. Half of the studies included in our review used this type of information in a model-based evaluation of interventions. A disease model is expected to incorporate as much data from the examined country [32] as possible, e.g. information on disease and strength and prevalence of risk factors, effectiveness of interventions within the population, the resource use needed, as well as prices for goods and services. Obviously, not all data is always available for all developing countries for reasons mentioned already above. This forces the scientist to transfer data from countries where this information is available to the country under analysis. Among those 18 studies that did use modeling in our review, none used external data on prices for services and goods, three used epidemiological and risk factor data from other countries, five used data on resource use from other settings, and all 18 studies used aggregated efficacy data from other healthcare settings to model the cost-effectiveness of interventions, without accounting for the presence of other risk factors. This shows that in particular data on effectiveness (and efficacy) of multiple and combined risk factor interventions is scarce for developing countries. In particular, large RCTs or meta-analyses of effectiveness studies are missing. Most estimates for effectiveness in those cases were based on data from developed countries. A study by Goeree et al. [33] confirms that this is a general obstacle when transferring data among regions. Goeree et al. analyzed 40 economic evaluations, which had tried to transfer results of studies to other geographic areas (not necessarily developed to developing countries). They developed a scoring system for the comprehensiveness of transferability (see Table 2 Modeling approaches based on the three most commonly advocated transferability factors).

**Table 2:** Modeling approaches based on the three most commonly advocated transferability factors (adopted from Goeree et al.2007 [33])

Modeling approach		Source of data by transferability factor		
		
Category		Clinical efficacy data	Resource utilization data	Unit cost data
Least to most country-specific analysis ↓	1	Studied country only	Studied country only	Mixture of studied and target country
	2	Studied country only	Studied country only	Target country only
	3	Studied country only	Mixture of studied and target country	Target country only
	4	Studied country only	Target country only	Target country only
	5	Target country only	Target country only	Target country only

While costs (in 39 out of 40 studies) and (some) resource use (28/40) was described as being most often adapted to the local setting, clinical efficacy was directly transferred from one geographic area to another. Only two out of 40 studies used efficacy data from the target country in their analysis. We applied the scoring system to the articles included in our sub-selection of modeled studies. Out of the 18 studies, none were *category 1*, six would be classified as *category 2*, two as *category 3*, seven as *category 4*, and none could fall into *category 5*. Three studies did not supply enough information for an explicit classification.

It can be expected that the effectiveness of risk factor and disease interventions will differ between developing and developed countries, given the often large differences in cultural, economic, infrastructural, and health care aspects and differences in risk factor epidemiology. In addition, biological differences may exist between ethnic groups [34], possibly based on pharmacogenetics, which might in some cases contribute to differences in the efficacy of certain drugs.

There are no specific guidelines on how to handle this type of uncertainty in modeling interventions for developing countries. While the guidelines for conducting a CEA in the methods section of the DCP2 acknowledges the lack of sufficient effectiveness data, no recommendations for transferring data from developed countries to the context of low resource settings is provided. One explanation suggests, however, that:

*"Besides the quality of the evidence at its source, how the results will apply to other settings matters, particularly when the data are limited to high-income countries. The more that outcomes depend on underlying biology, the more the findings will apply to low- and middle-income countries. Outcomes depending more on cultural or environmental factors are less readily transferred and require judgment and evidence as to their applicability elsewhere*." [16]

This may reflect the fact that many researchers believe or assume that clinical effects (in particular biological effects) of the interventions are transferable across health care systems but resource use and unit costs are more location specific. This is often common practice in HIC settings, while others tend to be more cautious in this respect: the WHO-CHOICE project developed in their guidelines a method on how to deal with differences in effectiveness. Effectiveness for developing countries is obtained by adjusting efficacy of clinical studies in developed countries by a factor between 0 and 1, based on the literature--or expert opinion as a last resort to account for these uncertainties [35]. Even though this method seems basic and its validity is not proven, it emphasizes the need to consider these influences in modeling [28].

As in all quantitative research, the use of valid and/or appropriate mathematical models is key when evaluating interventions in a generic way and for long-term [36]. Its critical assumptions may affect outcomes directly. The validity of the used results from randomized controlled trials for a particular study population may be limited as there can be a bias [37]. The impact of both the model and trial assumptions may be equally large. A thorough assessment of the models used in the reviewed papers, while desirable in principle, has on the whole not been possible due to the lack of detailed information on the precise model used in the studies. To allow more transparent analysis and appreciation of the modeling, we recommend, however, that future modeling analyses employ check-lists on good-modeling practices [27]. Unal et al. [38] already systematically assessed the quality of 42 different models on cardiovascular disease and stated that only 5 (12%) of them were comprehensive enough, considering both all relevant risk factors and types of treatments. The authors describe a vast variety in the quality and utility of mathematical models, in particular limited validation and calibration against observed data. Quality standards for modeling studies should be part of review processes (such as proposed by ISPOR [39]).

### Limitations

Our study has several limitations that need to be borne in mind when interpreting the results. First, our search strategy focused on cardiovascular disease treatment and its prevention. We did not single out certain risk factors to be included in the search string (as mentioned for tobacco). Therefore, some studies that did not mention CVD as intervention target might not be represented in our systematic review. Secondly, our systematic review is limited to a limited set of languages, i.e. German, French, Spanish, and Italian. In particular the growing literature in Chinese language, which is often not abstracted in English, is neglected by our search strategy. While recent and ongoing work of ours suggests that there is indeed a wealth of studies on the *effectiveness *of certain interventions targeting non-communicable diseases more generally published in the Chinese language, the locally published *cost-effectiveness *research remains scarce [40]. Nevertheless, we cannot completely exclude the possibility that the inclusion of more languages might critically affect our results.

## Conclusions

Even though the burden of cardiovascular disease in developing countries has been a neglected topic for decades, the evidence about cost-effective strategies to combat CVDs has been increasing since 2006, coinciding with the publication of the DCP2 report. Nevertheless, what economic evidence exists in low and middle income countries is valuable but scarce when compared to developed countries [5]. While the studies that we reviewed were biased towards individual-level interventions, mostly pharmaceutical, targeted at persons with already established risk factors, approaches that follow a population-based, non-clinical intervention strategy hardly appear to have been submitted to a cost-effectiveness analysis.

The presence of a majority of studies reporting the cost-effectiveness of different pharmaceutical interventions does not settle the debate as to whether the pharmaceutical approach is the main one to pursue, as no direct comparison to non-pharmaceutical interventions was made. The full evaluation of rolling out a pharmaceutical strategy would also need to consider the potentially prohibitive costs of scaling up screening for risk factors and the infrastructure of supplying drugs for identified persons and limited patient and system compliance--all factors that are hard to gauge particularly in developing countries. The experience in developed countries with extensive programmes to reduce risk factors through large-scale pharmaceutical interventions targeted at the high risk population for CVD, instead of preventing this risk factor from occurring by tackling the underlying causes, has increased the number of people requiring treatment. Providing medication for all of them is stretching health care budgets of many nations--as the example of hypertension shows [41].

A slight shift in the approach towards population-based non-clinical policies has been observed in developed countries like the United Kingdom or Finland. New strategies are applied to tackle the origin of the epidemics, such as legislations for salt reduction and food labeling [42]. As population-based interventions are being tried and tested, more evidence will hopefully emerge as to the true scope for them to make a difference to the CVD burden.

Our research also highlighted the most under-researched regions of the world in this context. Even though countries such as Armenia and Kazakhstan or Tunisia and Egypt suffer from a high burden of non-communicable diseases (around 60-80% of years of life lost (YLLs) [43]), those regions have not been the target of economic evaluations of CVD interventions. Promoting and incentivizing epidemiological and evaluation research in those regions should be part of an international health strategy on CVD risk reduction.

Another important insight concerns the data collection in developing countries. The methods used in the economic evaluations vary significantly and the adherence to common economic evaluation guidelines is less than widespread. These are the main reasons why a hierarchical listing of the study results is not feasible. A large share of the studies included in our review used mathematical modeling as a technique for economic evaluation. The reasons as well as the opportunities and limits have been described above. On one hand the scientific community ought to consider more explicitly how to handle the uncertainty (in effectiveness and compliance) associated with mathematical modeling. There is a need to identify affordable research designs as alternatives to either modeling with data from randomized controlled trials conducted in developed countries or directly conducting randomized controlled trials in developing regions. Other sources of data can contribute in important ways to the evidence base (e.g., demonstrating how a drug works in populations or under conditions usually not studied in the trial)--the idea of producing so-called "Real-World data" (RWD). The advantage of such effectiveness studies in informing the decision maker have been laid out in the discussion. A task force commissioned by the International Society for Pharmacoeconomics and Outcomes Research (ISPOR), identified the generation of RWD as a useful instrument to support RCTs, validate outcomes across different (sub-)populations or geographic regions, and to help with "sound coverage and reimbursement decisions" [44]. This data can be derived from large simple implementation trials, registry entries, or population health surveys, acknowledging the loss of evidence strength. It could well be a pragmatic and promising way forward in the urgent quest for the best buys in tackling CVDs.

Obviously, any cost-effectiveness evidence hinges on the presence of effectiveness evidence in the first place. In this report we have not searched explicitly for studies that focused on the sheer effectiveness of the intervention. However, there exist other recent efforts that have provided an overview of the evidence base on some of the interventions that had not undergone an economic evaluation. For example, a recent report published by the WHO [45] on interventions on diet and physical activity summarized all effectiveness evidence for different interventions, assessed the quality of the studies included and stated the degree of the effectiveness of interventions. A large variety of common-sense and promising, effective measures to improve diet and physical activity could be identified, including e.g. regulatory policies, point-of-decision prompts to encourage--for example--using stairs, or certain mass media campaigns. Such reports on "what works" are likely to represent a helpful first step in filling in the gaps in research on cost-effectiveness and may be used for further economic analysis in the future.

## Competing interests

The authors declare that they have no competing interests.

## Authors' contributions

MS, TAB and LN conceived of the study and participated in its design. MS and TAB conducted the literature review. TAB coordinated data retrieval, extraction and analysis. All authors participated in the interpretation of results and drafting of the manuscript. All authors read and approved the final manuscript.

## Pre-publication history

The pre-publication history for this paper can be accessed here:

http://www.biomedcentral.com/1471-2458/12/2/prepub

## Supplementary Material

Additional file 1 **PRISMA checklist for systematic reviews**. Click here for file

Additional file 2 **Search strings applied for the review**. Click here for file

Additional file 3 **Descriptive table of results**. Click here for file

Additional file 4 **Table of detailed results from included studies**. Click here for file
