# Supplementary material for: A systematic review of economic evaluations of interventions to tackle cardiovascular disease in low- and middle-income countries
Source: BMC Public Health. 2012 Jan 3;12:2. doi: 10.1186/1471-2458-12-2 (PMC3299641; doi:10.1186/1471-2458-12-2)
Supplement: Additional file 2 — Search strings applied for the review. [file 1471-2458-12-2-S2.DOC]

## Search strings applied for the review

**Search (PubMed) = 405 Articles**

("Cardiovascular Diseases"[Mesh] AND ("Costs and Cost Analysis"[Mesh] OR "Cost-effectiveness"[All Fields] OR "Cost-effective"[All Fields] OR "Cost-Utility"[All Fields] OR "Economic Evaluation"[All Fields] )) AND ("Developing Countries" OR "Afghanistan"[Mesh] OR "Bangladesh"[Mesh] OR "Benin"[Mesh] OR "Burkina Faso"[Mesh] OR "Burundi"[Mesh] OR "Cambodia"[Mesh] OR "Central African Republic"[Mesh] OR "Chad"[Mesh] OR "Comoros"[Mesh] OR "Democratic Republic of the Congo"[Mesh] OR "Cote d'Ivoire"[Mesh] OR "Eritrea"[Mesh] OR "Ethiopia"[Mesh] OR "Gambia"[Mesh] OR "Ghana"[Mesh] OR "Guinea"[Mesh] OR "Guinea-Bissau"[Mesh] OR "Haiti"[Mesh] OR "Kenya"[Mesh] OR "Korea"[Mesh] OR "Kyrgyzstan"[Mesh] OR "Laos"[Mesh] OR "Liberia"[Mesh] OR "Madagascar"[Mesh] OR "Malawi"[Mesh] OR "Mali"[Mesh] OR "Mauritania"[Mesh] OR "Mozambique"[Mesh] OR "Myanmar"[Mesh] OR "Nepal"[Mesh] OR "Niger"[Mesh] OR "Nigeria"[Mesh] OR "Pakistan"[Mesh] OR "Papua New Guinea"[Mesh] OR "Rwanda"[Mesh] OR "Atlantic Islands"[Mesh] OR "Senegal"[Mesh] OR "Sierra Leone"[Mesh] OR "Melanesia"[Mesh] OR "Somalia"[Mesh] OR "Tajikistan"[Mesh] OR "Tanzania"[Mesh] OR "Togo"[Mesh] OR "Uganda"[Mesh] OR "Uzbekistan"[Mesh] OR "Vietnam"[Mesh] OR "Yemen"[Mesh] OR "Zambia"[Mesh] OR "Zimbabwe"[Mesh] OR "Albania"[Mesh] OR "Algeria"[Mesh] OR "Angola"[Mesh] OR "Armenia"[Mesh] OR "Azerbaijan"[Mesh] OR "Bhutan"[Mesh] OR "Bolivia"[Mesh] OR "Bosnia-Herzegovina"[Mesh] OR "Cameroon"[Mesh] OR "Cape Verde"[Mesh] OR "China"[Mesh] OR "Colombia"[Mesh] OR "Congo"[Mesh] OR "Djibouti"[Mesh] OR "Dominican Republic"[Mesh] OR "Ecuador"[Mesh] OR "Egypt"[Mesh] OR "El Salvador"[Mesh] OR "Georgia (Republic)"[Mesh] OR "Guatemala"[Mesh] OR "Guyana"[Mesh] OR "Honduras"[Mesh] OR "India"[Mesh] OR "Indonesia"[Mesh] OR "Iran"[Mesh] OR "Iraq"[Mesh] OR "Jordan"[Mesh] OR "Micronesia"[Mesh] OR "Lesotho"[Mesh] OR "Macedonia (Republic)"[Mesh] OR "Indian Ocean Islands"[Mesh] OR "Micronesia"[Mesh] OR "Moldova"[Mesh] OR "Mongolia"[Mesh] OR "Morocco"[Mesh] OR "Namibia"[Mesh] OR "Nicaragua"[Mesh] OR "Paraguay"[Mesh] OR "Peru"[Mesh] OR "Philippines"[Mesh] OR "Samoa"[Mesh] OR "Sri Lanka"[Mesh] OR "Sudan"[Mesh] OR "Swaziland"[Mesh] OR "Syria"[Mesh] OR "Thailand"[Mesh] OR "East Timor"[Mesh] OR "Tonga"[Mesh] OR "Tunisia"[Mesh] OR "Turkmenistan"[Mesh] OR "Ukraine"[Mesh] OR "Vanuatu"[Mesh] OR "Tunisia"[Mesh] OR "Tunisia"[Mesh] OR "Tunisia"[Mesh] OR "Tunisia"[Mesh] OR "Tunisia"[Mesh] OR "American Samoa"[Mesh] OR "Argentina"[Mesh] OR "Byelarus"[Mesh] OR "Belize"[Mesh] OR "Botswana"[Mesh] OR "Brazil"[Mesh] OR "Bulgaria"[Mesh] OR "Chile"[Mesh] OR "Costa Rica"[Mesh] OR "Croatia"[Mesh] OR "Cuba"[Mesh] OR "Dominica"[Mesh] OR "Fiji"[Mesh] OR "Gabon"[Mesh] OR "Grenada"[Mesh] OR "Jamaica"[Mesh] OR "Kazakhstan"[Mesh] OR "Latvia"[Mesh] OR "Lebanon"[Mesh] OR "Libya"[Mesh] OR "Lithuania"[Mesh] OR "Malaysia"[Mesh] OR "Mauritius"[Mesh] OR "Comoros"[Mesh] OR "Mexico"[Mesh] OR "Montenegro"[Mesh] OR "Palau"[Mesh] OR "Panama"[Mesh] OR "Poland"[Mesh] OR "Romania"[Mesh] OR "Russia"[Mesh] OR "Serbia"[Mesh] OR "Seychelles"[Mesh] OR "South Africa"[Mesh] OR "Saint Kitts and Nevis"[Mesh] OR "Saint Lucia"[Mesh] OR "Saint Vincent and the Grenadines"[Mesh] OR "Suriname"[Mesh] OR "Turkey"[Mesh] OR "Uruguay"[Mesh] OR "Venezuela"[Mesh])

**Search (EconLit) = 198 Articles**

("cost benefit" or "cost effectiveness" or "economic evaluation" or "cost effective" or "cost utility" or "evaluation" or "cost minimization" or “cost efficiency” or “pharmacoeconomic”) and ("developing countr*" or "Afghanistan" OR "Bangladesh" OR "Benin" OR "Burkina Faso" OR "Burundi" OR "Cambodia" OR "Central African Republic" OR "Chad" OR "Comoros" OR "Democratic Republic of the Congo" OR "Cote d'Ivoire" OR "Eritrea" OR "Ethiopia" OR "Gambia" OR "Ghana" OR "Guinea" OR "Guinea-Bissau" OR "Haiti" OR "Kenya" OR "Korea" OR "Kyrgyzstan" OR "Laos" OR "Liberia" OR "Madagascar" OR "Malawi" OR "Mali" OR "Mauritania" OR "Mozambique" OR "Myanmar" OR "Nepal" OR "Niger" OR "Nigeria" OR "Pakistan" OR "Papua New Guinea" OR "Rwanda" OR "Atlantic Islands" OR "Senegal" OR "Sierra Leone" OR "Melanesia" OR "Somalia" OR "Tajikistan" OR "Tanzania" OR "Togo" OR "Uganda" OR "Uzbekistan" OR "Vietnam" OR "Yemen" OR "Zambia" OR "Zimbabwe" OR "Albania" OR "Algeria" OR "Angola" OR "Armenia" OR "Azerbaijan" OR "Bhutan" OR "Bolivia" OR "Bosnia-Herzegovina" OR "Cameroon" OR "Cape Verde" OR "China" OR "Colombia" OR "Congo" OR "Djibouti" OR "Dominican Republic" OR "Ecuador" OR "Egypt" OR "El Salvador" OR "Georgia" OR "Guatemala" OR "Guyana" OR "Honduras" OR "India" OR "Indonesia" OR "Iran" OR "Iraq" OR "Jordan" OR "Micronesia" OR "Lesotho" OR "Macedonia " OR "Indian Ocean Islands" OR "Micronesia" OR "Moldova" OR "Mongolia" OR "Morocco" OR "Namibia" OR "Nicaragua" OR "Paraguay" OR "Peru" OR "Philippines" OR "Samoa" OR "Sri Lanka" OR "Sudan" OR "Swaziland" OR "Syria" OR "Thailand" OR "East Timor" OR "Tonga" OR "Tunisia" OR "Turkmenistan" OR "Ukraine" OR "Vanuatu" OR "Tunisia" OR "American Samoa" OR "Argentina" OR "Belarus" OR "Belize" OR "Botswana" OR "Brazil" OR "Bulgaria" OR "Chile" OR "Costa Rica" OR "Croatia" OR "Cuba" OR "Dominica" OR "Fiji" OR "Gabon" OR "Grenada" OR "Jamaica" OR "Kazakhstan" OR "Latvia" OR "Lebanon" OR "Libya" OR "Lithuania" OR "Malaysia" OR "Mauritius" OR "Comoros" OR "Mexico" OR "Montenegro" OR "Palau" OR "Panama" OR "Poland" OR "Romania" OR "Russia" OR "Serbia" OR "Seychelles" OR "South Africa" OR "Saint Kitts and Nevis" OR "Saint Lucia" OR "Saint Vincent and the Grenadines" OR "Suriname" OR "Turkey" OR "Uruguay" OR "Venezuela") and "health"

**Search (Embase via DIMDI homepage) = 268 Articles**

1. EM74
2. CT="CARDIOVASCULAR DISEASE"
3. ((CT D "Cost Benefit analysis" OR UT="Cost Benefit analysis" OR IT="Cost Benefit analysis" OR SH="Cost Benefit analysis") OR (CT D "Economic Evaluation" OR UT="Economic Evaluation" OR IT="Economic Evaluation" OR SH="Economic Evaluation")) OR (CT D ("Cost effectiveness analysis"; "Cost Minimization analysis"; "Cost Utility analysis"; "Pharmacoeconomic analysis") OR UT=("Cost effectiveness analysis"; "Cost Minimization analysis"; "Cost Utility analysis"; "Pharmacoeconomic analysis") OR IT=("Cost effectiveness analysis"; "Cost Minimization analysis"; "Cost Utility analysis"; "Pharmacoeconomic analysis") OR SH=("Cost effectiveness analysis"; "Cost Minimization analysis"; "Cost Utility analysis"; "Pharmacoeconomic analysis"))
4. ((CT D "Developing Countries" OR UT="Developing Countries" OR IT="Developing Countries" OR SH="Developing Countries") OR (CT D ("Afghanistan"; "Bangladesh"; "Benin"; "Burkina Faso"; "Burundi"; "Cambodia"; "Central African Republic"; "Chad"; "Comos"; "Democratic Republic of the Congo"; "Cote d'Ivoire"; "Eritrea"; "Ethiopia"; "Gambia"; "Ghana"; "Guinea"; "Guinea-Bissau"; "Haiti"; "Kenya"; "North K ea"; "Kyrgyzstan"; "Laos"; "Liberia"; "Madagascar"; "Malawi"; "Mali"; "Mauritania"; "Mozambique"; "Myanmar"; "Nepal"; "Niger"; "Nigeria"; "Pakistan"; "Papua New Guinea"; "Rwanda"; "Atlantic Islands"; "Senegal"; "Sierra Leone"; "Melanesia"; "Somalia"; "Tajikistan"; "Tanzania"; "Togo"; "Uganda"; "Uzbekistan"; "Vietnam"; "Yemen"; "Zambia"; "Zimbabwe"; "Albania"; "Algeria"; "Angola"; "Armenia"; "Azerbaijan"; "Bhutan"; "Bolivia"; "Bosnia-Herzegovina"; "Cameroon"; "Cape Verde"; "China"; "Colombia"; "Congo"; "Djibouti"; "Dominican Republic"; "Ecuador"; "Egypt"; "El Salvador"; "Georgia") OR UT=("Afghanistan"; "Bangladesh"; "Benin"; "Burkina Faso"; "Burundi"; "Cambodia"; "Central African Republic"; "Chad"; "Comos"; "Democratic Republic of the Congo"; "Cote d'Ivoire"; "Eritrea"; "Ethiopia"; "Gambia"; "Ghana"; "Guinea"; "Guinea-Bissau"; "Haiti"; "Kenya"; "North K ea"; "Kyrgyzstan"; "Laos"; "Liberia"; "Madagascar"; "Malawi"; "Mali"; "Mauritania"; "Mozambique"; "Myanmar"; "Nepal"; "Niger"; "Nigeria"; "Pakistan"; "Papua New Guinea"; "Rwanda"; "Atlantic Islands"; "Senegal"; "Sierra Leone"; "Melanesia"; "Somalia"; "Tajikistan"; "Tanzania"; "Togo"; "Uganda"; "Uzbekistan"; "Vietnam"; "Yemen"; "Zambia"; "Zimbabwe"; "Albania"; "Algeria"; "Angola"; "Armenia"; "Azerbaijan"; "Bhutan"; "Bolivia"; "Bosnia-Herzegovina"; "Cameroon"; "Cape Verde"; "China"; "Colombia"; "Congo"; "Djibouti"; "Dominican Republic"; "Ecuador"; "Egypt"; "El Salvador"; "Georgia") OR IT=("Afghanistan"; "Bangladesh"; "Benin"; "Burkina Faso"; "Burundi"; "Cambodia"; "Central African Republic"; "Chad"; "Comos"; "Democratic Republic of the Congo"; "Cote d'Ivoire"; "Eritrea"; "Ethiopia"; "Gambia"; "Ghana"; "Guinea"; "Guinea-Bissau"; "Haiti"; "Kenya"; "North K ea"; "Kyrgyzstan"; "Laos"; "Liberia"; "Madagascar"; "Malawi"; "Mali"; "Mauritania"; "Mozambique"; "Myanmar"; "Nepal"; "Niger"; "Nigeria"; "Pakistan"; "Papua New Guinea"; "Rwanda"; "Atlantic Islands"; "Senegal"; "Sierra Leone"; "Melanesia"; "Somalia"; "Tajikistan"; "Tanzania"; "Togo"; "Uganda"; "Uzbekistan"; "Vietnam"; "Yemen"; "Zambia"; "Zimbabwe"; "Albania"; "Algeria"; "Angola"; "Armenia"; "Azerbaijan"; "Bhutan"; "Bolivia"; "Bosnia-Herzegovina"; "Cameroon"; "Cape Verde"; "China"; "Colombia"; "Congo"; "Djibouti"; "Dominican Republic"; "Ecuador"; "Egypt"; "El Salvador"; "Georgia") OR SH=("Afghanistan"; "Bangladesh"; "Benin"; "Burkina Faso"; "Burundi"; "Cambodia"; "Central African Republic"; "Chad"; "Comos"; "Democratic Republic of the Congo"; "Cote d'Ivoire"; "Eritrea"; "Ethiopia"; "Gambia"; "Ghana"; "Guinea"; "Guinea-Bissau"; "Haiti"; "Kenya"; "North K ea"; "Kyrgyzstan"; "Laos"; "Liberia"; "Madagascar"; "Malawi"; "Mali"; "Mauritania"; "Mozambique"; "Myanmar"; "Nepal"; "Niger"; "Nigeria"; "Pakistan"; "Papua New Guinea"; "Rwanda"; "Atlantic Islands"; "Senegal"; "Sierra Leone"; "Melanesia"; "Somalia"; "Tajikistan"; "Tanzania"; "Togo"; "Uganda"; "Uzbekistan"; "Vietnam"; "Yemen"; "Zambia"; "Zimbabwe"; "Albania"; "Algeria"; "Angola"; "Armenia"; "Azerbaijan"; "Bhutan"; "Bolivia"; "Bosnia-Herzegovina"; "Cameroon"; "Cape Verde"; "China"; "Colombia"; "Congo"; "Djibouti"; "Dominican Republic"; "Ecuador"; "Egypt"; "El Salvador"; "Georgia"))) OR (CT D ("Guatemala"; "Guyana"; "Honduras"; "India"; "Indonesia"; "Iran"; "Iraq"; "Jordan"; "Micronesia"; "Lesotho"; "Macedonia"; "Indian Ocean Islands"; "Micronesia"; "Moldova"; "Mongolia"; "Morocco"; "Namibia"; "Nicaragua"; "Paraguay"; "Peru"; "Philippines"; "Samoa"; "Sri Lanka"; "Sudan"; "Swaziland"; "Syria"; "Thailand"; "East Timor"; "Tonga"; "Tunisia"; "Turkmenistan"; "Ukraine"; "Vanuatu"; "Tunisia"; "Tunisia"; "Tunisia"; "Tunisia"; "Tunisia"; "American Samoa"; "Argentina"; "Belarus"; "Belize"; "Botswana"; "Brazil"; "Bulgaria"; "Chile"; "Costa Rica"; "Croatia"; "Cuba"; "Dominica"; "Fiji"; "Gabon"; "Grenada"; "Jamaica"; "Kazakhstan"; "Latvia"; "Lebanon"; "Libya"; "Lithuania"; "Malaysia"; "Mauritius"; "Comoros"; "Mexico"; "Montenegro"; "Palau"; "Panama"; "Poland"; "Romania"; "Russia"; "Serbia"; "Seychelles"; "South Africa"; "Saint Kitts and Nevis"; "Saint Lucia"; "Saint Vincent and the Grenadines"; "Suriname"; "Turkey"; "Uruguay"; "Venezuela") OR UT=("Guatemala"; "Guyana"; "Honduras"; "India"; "Indonesia"; "Iran"; "Iraq"; "Jordan"; "Micronesia"; "Lesotho"; "Macedonia"; "Indian Ocean Islands"; "Micronesia"; "Moldova"; "Mongolia"; "Morocco"; "Namibia"; "Nicaragua"; "Paraguay"; "Peru"; "Philippines"; "Samoa"; "Sri Lanka"; "Sudan"; "Swaziland"; "Syria"; "Thailand"; "East Timor"; "Tonga"; "Tunisia"; "Turkmenistan"; "Ukraine"; "Vanuatu"; "Tunisia"; "Tunisia"; "Tunisia"; "Tunisia"; "Tunisia"; "American Samoa"; "Argentina"; "Belarus"; "Belize"; "Botswana"; "Brazil"; "Bulgaria"; "Chile"; "Costa Rica"; "Croatia"; "Cuba"; "Dominica"; "Fiji"; "Gabon"; "Grenada"; "Jamaica"; "Kazakhstan"; "Latvia"; "Lebanon"; "Libya"; "Lithuania"; "Malaysia"; "Mauritius"; "Comoros"; "Mexico"; "Montenegro"; "Palau"; "Panama"; "Poland"; "Romania"; "Russia"; "Serbia"; "Seychelles"; "South Africa"; "Saint Kitts and Nevis"; "Saint Lucia"; "Saint Vincent and the Grenadines"; "Suriname"; "Turkey"; "Uruguay"; "Venezuela") OR IT=("Guatemala"; "Guyana"; "Honduras"; "India"; "Indonesia"; "Iran"; "Iraq"; "Jordan"; "Micronesia"; "Lesotho"; "Macedonia"; "Indian Ocean Islands"; "Micronesia"; "Moldova"; "Mongolia"; "Morocco"; "Namibia"; "Nicaragua"; "Paraguay"; "Peru"; "Philippines"; "Samoa"; "Sri Lanka"; "Sudan"; "Swaziland"; "Syria"; "Thailand"; "East Timor"; "Tonga"; "Tunisia"; "Turkmenistan"; "Ukraine"; "Vanuatu"; "Tunisia"; "Tunisia"; "Tunisia"; "Tunisia"; "Tunisia"; "American Samoa"; "Argentina"; "Belarus"; "Belize"; "Botswana"; "Brazil"; "Bulgaria"; "Chile"; "Costa Rica"; "Croatia"; "Cuba"; "Dominica"; "Fiji"; "Gabon"; "Grenada"; "Jamaica"; "Kazakhstan"; "Latvia"; "Lebanon"; "Libya"; "Lithuania"; "Malaysia"; "Mauritius"; "Comoros"; "Mexico"; "Montenegro"; "Palau"; "Panama"; "Poland"; "Romania"; "Russia"; "Serbia"; "Seychelles"; "South Africa"; "Saint Kitts and Nevis"; "Saint Lucia"; "Saint Vincent and the Grenadines"; "Suriname"; "Turkey"; "Uruguay"; "Venezuela") OR SH=("Guatemala"; "Guyana"; "Honduras"; "India"; "Indonesia"; "Iran"; "Iraq"; "Jordan"; "Micronesia"; "Lesotho"; "Macedonia"; "Indian Ocean Islands"; "Micronesia"; "Moldova"; "Mongolia"; "Morocco"; "Namibia"; "Nicaragua"; "Paraguay"; "Peru"; "Philippines"; "Samoa"; "Sri Lanka"; "Sudan"; "Swaziland"; "Syria"; "Thailand"; "East Timor"; "Tonga"; "Tunisia"; "Turkmenistan"; "Ukraine"; "Vanuatu"; "Tunisia"; "Tunisia"; "Tunisia"; "Tunisia"; "Tunisia"; "American Samoa"; "Argentina"; "Belarus"; "Belize"; "Botswana"; "Brazil"; "Bulgaria"; "Chile"; "Costa Rica"; "Croatia"; "Cuba"; "Dominica"; "Fiji"; "Gabon"; "Grenada"; "Jamaica"; "Kazakhstan"; "Latvia"; "Lebanon"; "Libya"; "Lithuania"; "Malaysia"; "Mauritius"; "Comoros"; "Mexico"; "Montenegro"; "Palau"; "Panama"; "Poland"; "Romania"; "Russia"; "Serbia"; "Seychelles"; "South Africa"; "Saint Kitts and Nevis"; "Saint Lucia"; "Saint Vincent and the Grenadines"; "Suriname"; "Turkey"; "Uruguay"; "Venezuela"))
5. 2 AND 3 AND 4

**Search (NHS EED) = 82 Articles**

“Cardiovascular Disease*” AND (“developing countr*" OR "Afghanistan" OR "Bangladesh" OR "Benin" OR "Burkina Faso" OR "Burundi" OR "Cambodia" OR "Central African Republic" OR "Chad" OR "Comoros" OR "Democratic Republic of the Congo" OR "Cote d'Ivoire" OR "Eritrea" OR "Ethiopia" OR "Gambia" OR "Ghana" OR "Guinea" OR "Guinea-Bissau" OR "Haiti" OR "Kenya" OR "Korea" OR "Kyrgyzstan" OR "Laos" OR "Liberia" OR "Madagascar" OR "Malawi" OR "Mali" OR "Mauritania" OR "Mozambique" OR "Myanmar" OR "Nepal" OR "Niger" OR "Nigeria" OR "Pakistan" OR "Papua New Guinea" OR "Rwanda" OR "Atlantic Islands" OR "Senegal" OR "Sierra Leone" OR "Melanesia" OR "Somalia" OR "Tajikistan" OR "Tanzania" OR "Togo" OR "Uganda" OR "Uzbekistan" OR "Vietnam" OR "Yemen" OR "Zambia" OR "Zimbabwe" OR "Albania" OR "Algeria" OR "Angola" OR "Armenia" OR "Azerbaijan" OR "Bhutan" OR "Bolivia" OR "Bosnia-Herzegovina" OR "Cameroon" OR "Cape Verde" OR "China" OR "Colombia" OR "Congo" OR "Djibouti" OR "Dominican Republic" OR "Ecuador" OR "Egypt" OR "El Salvador" OR "Georgia" OR "Guatemala" OR "Guyana" OR "Honduras" OR "India" OR "Indonesia" OR "Iran" OR "Iraq" OR "Jordan" OR "Micronesia" OR "Lesotho" OR "Macedonia " OR "Indian Ocean Islands" OR "Micronesia" OR "Moldova" OR "Mongolia" OR "Morocco" OR "Namibia" OR "Nicaragua" OR "Paraguay" OR "Peru" OR "Philippines" OR "Samoa" OR "Sri Lanka" OR "Sudan" OR "Swaziland" OR "Syria" OR "Thailand" OR "East Timor" OR "Tonga" OR "Tunisia" OR "Turkmenistan" OR "Ukraine" OR "Vanuatu" OR "Tunisia" OR "American Samoa" OR "Argentina" OR "Belarus" OR "Belize" OR "Botswana" OR "Brazil" OR "Bulgaria" OR "Chile" OR "Costa Rica" OR "Croatia" OR "Cuba" OR "Dominica" OR "Fiji" OR "Gabon" OR "Grenada" OR "Jamaica" OR "Kazakhstan" OR "Latvia" OR "Lebanon" OR "Libya" OR "Lithuania" OR "Malaysia" OR "Mauritius" OR "Comoros" OR "Mexico" OR "Montenegro" OR "Palau" OR "Panama" OR "Poland" OR "Romania" OR "Russia" OR "Serbia" OR "Seychelles" OR "South Africa" OR "Saint Kitts and Nevis" OR "Saint Lucia" OR "Saint Vincent and the Grenadines" OR "Suriname" OR "Turkey" OR "Uruguay" OR "Venezuela")

*(Please note that Poland was dropped from search at a later stage of the review)*
