# Supplementary material for: A systematic review of economic evaluations of interventions to tackle cardiovascular disease in low- and middle-income countries
Source: BMC Public Health. 2012 Jan 3;12:2. doi: 10.1186/1471-2458-12-2 (PMC3299641; doi:10.1186/1471-2458-12-2)
Supplement: Additional file 3 — Descriptive table of results. [file 1471-2458-12-2-S3.DOC]

## Table of descriptive results

| **Category** | | **Pub Year**  **1989-2005** | **Pub Year**  **2006-2009** | **Total**  **(n=33)** |
| --- | --- | --- | --- | --- |
| Study Setting | | | | |
|  | Single Nation | 10 | 17 | 27 (82%) |
|  | Multi-National | 3 | 3 | 6 (18%) |
| Type of economic Evaluation | | | | |
|  | Cost-Consequence (CCA) | 4 | 6 | 10 (30%) |
|  | Cost-Effectiveness „Clinical“ | 5 | 5 | 10 (30%) |
|  | Cost-Utility (CUA) | 3 | 5 | 8 (24%) |
|  | Cost-Effectiveness „Life Years“ | 0 | 4 | 4 (12%) |
|  | Cost Minimization (CMA) | 1 | 0 | 1 (3%) |
|  | Cost-Benefit (CBA) | 0 | 0 | 0 (0%) |
| Type of Study for data generation | | | | |
|  | Modeling | 5 | 13 | 18 (55%) |
|  | Observational Trial | 3 | 4 | 7 (21%) |
|  | Randomized Controlled Trial(RCT) | 3 | 1 | 4 (12%) |
|  | Other* | 2 | 2 | 4 (12%) |
| Intervention Target | | | | |
|  | Risk Factor | 9 | 15 | 24 (73%) |
|  | Revascularization | 3 | 3 | 6 (18%) |
|  | Various | 1 | 1 | 2 (6%) |
|  | Other* | 0 | 1 | 1(3%) |
| Targeted Risk Factor | | | | |
|  | High Blood Pressure | 5 | 4 | 9 (27%) |
|  | Atrial Fibrillation | 0 | 1 | 1 (3%) |
|  | Blood Glucose | 0 | 0 | 0 (0%) |
|  | Smoking | 0 | 1 | 1 (3%) |
|  | Physical Inactivity | 0 | 1 | 1 (3%) |
|  | Dietary Intake | 0 | 0 | 0 (0%) |
|  | Dyslipidemia | 0 | 1 | 1 (3%) |
|  | Multiple | 4 | 7 | 11 (33%) |
|  | No Risk Factor analyzed | 4 | 5 | 9 (27%) |
| Type of Intervention | | | | |
|  | Case Management | 3 | 5 | 8 (24%) |
|  | Primary Prevention | 4 | 7 | 11 (33%) |
|  | Secondary Prevention | 1 | 4 | 5 (15%) |
|  | Various | 5 | 4 | 9 (27%) |
| Type of Intervention Measure | | | | |
|  | Pharmaceutical | 5 | 9 | 14 (42%) |
|  | Procedure | 3 | 4 | 7 (21%) |
|  | Health Education (Personal Level) | 1 | 2 | 3 (9%) |
|  | Social Marketing (Pop. based) | 1 | 1 | 2 (6%) |
|  | Medical Technology | 0 | 1 | 1 (3%) |
|  | Health Care Delivery | 1 | 1 | 2 (6%) |
|  | Various | 2 | 2 | 4 (12%) |
| * Summarized over different categories | | | | |
|  | | | | |
|  | | | | |
|  | | | | |
|  | | | | |
| **Category** | | **Pub Year**  **1989-2005** | **Pub Year**  **2006-2009** | **Total**  **(n=33)** |
| Source of Funding (as stated by the author) | | | | |
|  | Foundation | 2 | 1 | 3 (9%) |
|  | Government | 0 | 5 | 5 (15%) |
|  | Industry | 1 | 2 | 3 (9%) |
|  | Other* | 2 | 1 | 3 (9%) |
|  | Not stated | 8 | 11 | 19 (58%) |
| Economic Perspective (as explicitly stated by the author) | | | | |
|  | Health Care Sector | 3 | 4 | 7 (21%) |
|  | Health Insurance | 3 | 6 | 9 (27%) |
|  | Patient | 1 | 1 | 2 (6%) |
|  | Societal | 0 | 0 | 0 (0%) |
|  | Not stated | 6 | 9 | 15 (45%) |
| * Summarized over different categories | | | | |

| **Category** | | **Pub Year**  **1989-2005** | **Pub Year**  **2006-2009** | **Total**  **(n=11)** |
| --- | --- | --- | --- | --- |
| Intervention type *Primary Prevention* by target group | | | | |
|  | Personal intervention | 2 | 4 | 6 (55%) |
|  | Population based | 1 | 2 | 3 (27%) |
|  | Both (Mixed Strategy) | 1 | 1 | 2 (18%) |

| **Category** | | **Pub Year**  **1989-2005** | **Pub Year**  **2006-2009** | **Total**  **(n=8)** |
| --- | --- | --- | --- | --- |
| Intervention type *Case Management* in detail | | | | |
|  | Treatment* | 3 | 4 | 7 (88%) |
|  | Rehabilitation | 0 | 1 | 1 (12%) |
|  | Screening | 0 | 0 | 0 (0%) |
| * including Acute Care, Care, Chronic Care | | | | |
