# Supplementary material for: A systematic review of economic evaluations of interventions to tackle cardiovascular disease in low- and middle-income countries
Source: BMC Public Health. 2012 Jan 3;12:2. doi: 10.1186/1471-2458-12-2 (PMC3299641; doi:10.1186/1471-2458-12-2)
Supplement: Additional file 4 — Table of detailed results from included studies. [file 1471-2458-12-2-S4.DOC]

## *Table of detailed results from included studies 1/2 (Empirical studies) – please note, price levels are not standardized*

| **Primary author** | **Moreira, GC** | **Hongli Jiang, WC** | **Salvetti, X. M.** |
| --- | --- | --- | --- |
| **Pub Year** | 2009 | 2009 | 2008 |
| **Title** | Evaluation of the awareness, control and cost-effectiveness of hypertension treatment in a Brazilian city: populational study | Are services delivered by community health centers more cost-effective? Evidence from urban China | How much do the benefits cost? Effects of a home-based training programme on cardiovascular fitness, quality of life, programme cost and adherence for patients with coronary disease |
| **Journal** | Journal of Hypertension | Health Economics | Clin.Rehabil. |
| **Type of study** | CEA – CL | CEA – CL | CCA |
| **Study Setting** | Single Nation:  Brazil | Single Nation:  China | Single Nation:  Brazil |
| **Data generation** | Other:  Cross-sectional study | Observational Trial | RCT |
| **Size of a) intervention and b) control group in study** | a) ACEI n=106  b) Diuretics: n=64  ß-Blockers: n=26  AT-1 blocker, Calcium blocker, alpha-adrenergic blockers: n=26  Multiple agents: n=304 | a) Patients in CHCs: n=2147  b) Patients in Secondary and Tertiary hospitals: n=1092 | a) Home exercise: n=19  b) Control group: n=20 |
| **Intervention Target** | Risk factors  High Blood Pressure | Risk factor:  Various | Risk factor:  Physical Inactivity |
| **Type of Intervention** | Various  Primary and Secondary Prevention | Various  (Prim. & Sec. Prevention) | Case Management:  Rehabilitation |
| **Type of measure** | Pharmaceutical | Health Care Delivery | Health Education |
| **Benefit measure** | Blood Pressure | Blood Pressure/ Blood Glucose level | Quality of Life |
| **Funding source** | Not stated | Foundation | Not stated |
| **Economic perspective** | Not stated | Not stated | Not stated |
| **Study length and follow-up** | Only cross-sectional review, no follow-up | Not specified | 3 months intervention; no follow up reported |
| **Target Group:**  **1. Risk factor/Co-morbidity**  **2. Male/Female ratio**  **3. Age**  **4. Socioeconomic Status**  **5. Other underlying factors** | 1. Multiple 2. 47/53 3. 40->70 4. AB:~20%   C: ~41%  DE:~40%   1. Schooling, BMI | 1. Multiple co-morbidities, not specified  2. (34/66) & (39/61)  3. Mean: 69.8+/-9.6 & 70.2+/-9.0)  4. Not stated | 1. Post MI, Hypertension, Dylipidemia, Diabetes 2. Female/Male (25/75) 3. 54 +/- 9 4. First School Diploma: 90% 5. All from Sao Paolo, very strict inclusion criteria |
| **Intervention (detail)** | Treatment of hypertension with different antihypertensive agents in monotherapy or combination | Health care delivery (primary care) for hypertension and diabetes through community health centers (CHCs) in China | To evaluate cost, adherence and effects on cardiovascular function and  quality of life of a home-based cardiac rehabilitation programme for patients with  coronary disease.The home group performed home-based training for three months  with biweekly telephone monitoring. |
| **Comparator (detail)** | Treatment of hypertension with different antihypertensive agents in monotherapy or combination | Health care delivery through secondary care hospitals (ambulatory care) | Standard care |
| **Results (detail)** | Controlled Blood Pressure:  ß-Blocker: 66.4%  Diuretics: 56.6%  ACEI: 44.8%  ACEI+Diuretics: 54.7%  Diuretics+ß-Blocker: 67.9%  Cost-Effectiveness (Monotherapy) as in  Mean monthly value:  Diuretics: $15.5  ß-blockers: $34.7  ACEI: $176.7 | There was no significant difference in clinical health outcome, between CHCs and hospitals  Daily health expenditure was 2.14-5.90 RMB higher at hospitals than at CHCs | The home group had significant improvements inall domains of the SF-36. Control group showed improvement in only three of the eight domains and decline in the other five domains. The low-cost protocol costs  $502.71 (BHCMP) per patient for three months |
| **Use of discounting** | Notne stated | None stated | None Stated |

| **Primary author** | **Getpreechaswas, J.** | **Amira, C. O.** | **Diaz, T., V** |
| --- | --- | --- | --- |
| **Pub Year** | 2007 | 2006 | 2006 |
| **Title** | A model of health services for hypertension in primary care unit in Patumthani Province | Antihypertensive pharmacotherapy in a developing economy: pattern, acquisition costs and conformity to international guidelines in a tertiary-care setting | [Evaluation of a stroke unit at a university hospital in Chile] |
| **Journal** | J.Med.Assoc.Thai. | J.Hum.Hypertens. | Rev.Med.Chil. |
| **Type of study** | CEA-CL | CEA-CL | CCA |
| **Study Setting** | Single Nation:  Thailand | Single Nation:  Nigeria | Single Nation:  Chile |
| **Data generation** | Observational Trial | Other | Observational Trial |
| **Size of a) intervention and b) control group in study** | a) Group I: n=139  b) Group II: n=159  Group III: n=167 | a) CCB: n=16  b) DV: n= 8  b-blockers : n=8  ACEI : n=10  a-MD : n=8  Reserpine+clopamide : n=8  DV+a-MD : n=21  DV+CCB : n=24  DV+b-blockers : n=8  CCB+ACEI : n=24  CCB+b-blockers: n= 13  CCB+a-MD: n= 8  DV+CCB+b-blockers: n= 12  DV+CCB+a-MD: n= 9  CCB+ACEI+a-MD: n= 11  Abbreviations: ACEI, angiotensin-converting enzyme inhibitor; a-MD, a-methyldopa; CCB, calcium channel blocker; CER, cost-effectiveness ratio; DV, diuretic. | a) Stroke unit n=105  b) Hospital n=425 |
| **Intervention Target** | Risk factor:  High Blood Pressure | Risk factor:  High Blood Pressure | Revascularization |
| **Type of Intervention** | Primary Prevention:  Population Based | Various:  Personal Intervention | Case Management:  Treatment |
| **Type of measure** | Social marketing | Pharmaceutical | Procedure |
| **Benefit measure** | Behavior Change | Blood Pressure | Length of Stay |
| **Funding source** | Not stated | Not stated | Not stated |
| **Economic perspective** | Not stated | Patient | Health Care Sector |
| **Study length and follow-up** | Part I: August 2002 – July 2003  Part II: October – December 2004  Part III: November 2005 – April 2006 | 12 weeks cross-sectional study | 1 year observation, no follow-up period |
| **Target Group:**  **1. Risk factor/Co-morbidity**  **2. Male/Female ratio**  **3. Age**  **4. Socioeconomic Status**  **5. Other underlying factors** | 1. General Population 2. 18-59 3. Female/Male (~64/36) 4. Significant differences in education and income level between Intervention (high) and Control Group (low) | 1. High Blood Pressure, comorbidities in 31.6% (diabetes, previous heart condition, previous cerebrovascular disease) 2. Female/Male (60/40) 3. 55.1 ± 12.4 years 4. 3 Income Groups: Low($1-$150) (66.7%), Middle($150-$1500) (32.9%), High(>$1500) (.4%) 5. 225 Black individuals- All attended same tertiary hypertension clinic | 1. Stroke 2. Female/Male (58/42) 3. 30-89 years 4. Not stated |
| **Intervention (detail)** | To develop a model that utilized health personnel, village health volunteers (VHVs) and family health leaders (FHLD to improve health services to prevent and control hypertension in three primary care units (PCU) in Patumthani Province. The model was designed to include  two intervention groups (Group I, II) and a control group (Group III). The Group I was taught by trained health personnel, VHVs and FHLs. The Group II was taught by trained health personnel and VHVs. | Primary Prevention: 81 mg aspirin, 40 mg lovastatin, 10 mg lisinopril, and 5 mg amlodipine; Secondary Prevention: 81 mg aspirin, 40 mg lovastatin, 10 mg lisinopril, and 5 mg metoprolol | Care through stroke unit |
| **Comparator (detail)** | The Group III was interviewed only by trained health personnel. | Alternative drug | Regular hospital care |
| **Results (detail)** | Results from the  cost effectiveness study in Part II showed that the unit  cost per head of population, as adjusted for the level of behavior change, for Group I is the lowest, 74.89 Baht | ICER: primary prevention, US$746–890/QALY gained for patients with a 10-year absolute risk of cardiovascular disease greater than 25%, and $1039–1221/QALY gained for those with an absolute risk greater than 5%. Secondary prevention ranged from $306/QALY to $388/QALY gained | Stroke Unit: Mean length of stay: 6.6 days, mean cost per patient: US$ 5.550; Hospital: Mean length of stay: 9.9 days, mean cost per patient US$ 4.815 |
| **Use of discounting** | None stated | 3% per year | None stated |

| **Primary author** | **Vida, V. L.** | **Dias da Costa, J. S.** | **Garcia-Pena, C.** |
| --- | --- | --- | --- |
| **Pub Year** | 2006 | 2002 | 2002 |
| **Title** | Radiofrequency catheter ablation of supraventricular tachycardia in children and adolescents : feasibility and cost-effectiveness in a low-income country | Cost-effectiveness of hypertension treatment: a population-based study | Economic analysis of a pragmatic randomised trial of home visits by a nurse to elderly people with hypertension in Mexico |
| **Journal** | Pediatr.Cardiol. | Sao Paulo Med.J. | Salud Publica Mex. |
| **Type of study** | CCA | CEA-CL | CEA-CL |
| **Study Setting** | Single Nation:  Guatemala | Single Nation:  Brazil | Single Nation:  Mexico |
| **Data generation** | Observational Trial | Other | RCT |
| **Size of a) intervention and b) control group in study** | a) Radiofrequency catheter ablation: n=28  b) Hypothetical Control group – same group if they were not undergone therapy: n=28 | Diuretics: n=71  Beta blockers : n=31  Calcium channel blockers : n=10  ACE inhibitors : n=25  Diuretics + Beta blockers : n=36  Diuretics + Calcium channel blockers : n=13  Diuretics + ACE Inhibitors : n=30  Beta blockers + Calcium channel blockers : n=6  Beta blockers + ACE Inhibitors : n=3  Other combinations : n=34 | a) nurse visits: n=345  b) control group: n=338 |
| **Intervention Target** | Risk factor:  Atrial Fibrillation | Risk factor:  High Blood Pressure | Risk factor:  High Blood Pressure |
| **Type of Intervention** | Case Management:  Treatment | Secondary Prevention:  Personal Intervention | Various:  Personal Intervention |
| **Type of measure** | Procedure | Pharmaceutical | Health Education |
| **Benefit measure** | Other | Blood Pressure | Blood Pressure |
| **Funding source** | Not stated | Foundation | Not stated |
| **Economic perspective** | Health Care Sector | Patient | Health Insurance |
| **Study length and follow-up** | Average time of 13.69 months of follow-up (+/- 7.16 months)  Calculation of cost-effectiveness for hypothetical time period of 5 and 20 years | Real-life cross-sectional collection of data; retrospective costs analysis; no follow-up with patients reported | 7 months of intervention, no further follow up beyond this period reported |
| **Target Group:**  **1. Risk factor/Co-morbidity**  **2. Male/Female ratio**  **3. Age**  **4. Socioeconomic Status**  **5. Other underlying factors** | 1. Presence of paroxysmal supraventricular tachycardia (SVT) 2. Female/Male (29/71) 3. 11.42 ± 3.49 years 4. Not stated | 1. Hypertension, some with comorbidities (diabetes or smoking) 2. Female/Male (73/27) 3. 52.5 ± 10.5 years 4. Not Stated | 1. Hypertension 2. Female/Male (65/35) 3. 60 years and older 4. 55.6% had monthly income of $10-$109; 21.5% between $110-$209; and 4% >$210 |
| **Intervention (detail)** | Radiofrequency catheter ablation (RFCA) | Antihypertensives (diuretics, beta-blockers, and ACE inhibitors) | Visits to their home bi-weekly by a nurse giving health and lifestyle counseling |
| **Comparator (detail)** | Hypothetical continued medical treatment | Alternative drugs | Doing nothing |
| **Results (detail)**  **Results (continued)** | Mean cost per procedure(RFCA) was $1,668.1 +/- 357.08; estimated cost of RFCA will be equal that of continued medical therapy after 5.1 years and will be 3.4 times less than medical therapy after 20 years | Cost effectiveness ratio for each drug/drug combination: of monotherapies best was diuretics, CER 116.3, worst was calcium channel blockers, CER 762 | cost-effectiveness ratios was 10.46 pesos (US$1.14) for systolic (CI 95% 129.31, 5.51) and 9.43 (US$1.03) for diastolic (CI 95% 19.90, 2.49) |
| **Use of discounting** | None stated | None stated | 3% per year |

| **Primary author** | **Pannarunothai, S.** | **Edwards, P. R.** | **Grines, C. L.** |
| --- | --- | --- | --- |
| **Pub Year** | 2001 | 1998 | 1998 |
| **Title** | Costs-effectiveness of the urban health center in Nakhon Ratchasima: a case study on diabetes and hypertension | Improving cost-effectiveness of hypertension management at a community health centre | Safety and cost-effectiveness of early discharge after primary angioplasty in low risk patients with acute myocardial infarction. PAMI-II Investigators. Primary Angioplasty in Myocardial Infarction |
| **Journal** | J.Med.Assoc.Thai. | S.Afr.Med.J. | J.Am.Coll.Cardiol. |
| **Type of study** | CEA-CL | CMA | CCA |
| **Study Setting** | Single Nation:  Thailand | Single Nation:  South Africa | Multi-National:  Cross Country - Developed and Developing nations |
| **Data generation** | Observational Trial | Observational Trial | RCT |
| **Size of a) intervention and b) control group in study** | a) Urban health center: Diabetes n=36; Hypertension n=21  b) Regional Hospital (same area): Diabetes  n=24; Hypertension n=24  Regional Hospital (other area): Diabetes  n=37; Hypertension n=36 | a) Patients under study before and after intervention n=1084  b) Hypothetical group of patients remaining on current drug treatment | a) Accelerated care: n=237  b)Traditional care: n=234 |
| **Intervention Target** | Risk factor:  High Blood Pressure/Diabetes | Risk factor:  High Blood Pressure | Revascularization |
| **Type of Intervention** | Primary Prevention:  Personal Intervention | Various:  Personal Intervention | Case Management:  Treatment |
| **Type of measure** | Health Care Delivery | Pharmaceutical | Procedure |
| **Benefit measure** | Blood Press./ Blood Glucose | Blood Pressure | Avoided CVD incidents |
| **Funding source** | Other | Other | Not stated |
| **Economic perspective** | Health Care Sector | Health Care Sector | Not stated |
| **Study length and follow-up** | Recruiting period of two years; Costs calculated for one year; no explicit average follow-up period reported | 1 year intervention period; no further follow-up period reported | 6 months follow-up after intervention to measure primary end-point |
| **Target Group:**  **1. Risk factor/Co-morbidity**  **2. Male/Female ratio**  **3. Age**  **4. Socioeconomic Status**  **5. Other underlying factors** | 1. Hypertension or Diabetes 2. Not stated 3. Not stated 4. Not stated | 1. Hypertension, already being treated with prescriptions 2. Not stated 3. Not stated 4. Not stated 5. All from the same clinic in Cape Town | 1. Emergency catheterization following acute MI 2. Accelerated= Female/Male (22.4/77.6); Traditional = Female/Male (24.8/75.2) 3. Accelerated Care = 55 ± 10; Traditional = 56 ± 10 |
| **Intervention (detail)** | Healthcare delivery by a  a) Urban Health Center | 1. Implementation of stepped-care guidelines for hypertension, specifying treatment with more cost-effective drugs and minimising drug treatment. 2. Reduction of availability for routine prescribing of 10 less cost-effective antihypertensive drugs or drug combinations. | Accelerated care: admission to a nonintensive care unit and day 3 hospital discharge without noninvasive testing |
| **Comparator (detail)** | Healthcare delivery by  b) Regional Hospital (same area)  c) Regional Hospital (other area) | Current drug treatment | Traditional Care |
| **Results (detail)** | Total costs (baht) per % of effective treatment (defined by patient within recommended biomarker range)  Blood Pressure:   1. 5,729 2. 7,137 3. 7,195   Diabetes:   1. 7,468 2. 12,313 3. 17,861 | Significant decrease in drugs per patient due to rise in number of patients receiving no treatment; significant decrease in cost per patient due to decrease in prescriptions of "restricted" drugs | Accelerated care patients had similar in hospital outcomes and secondary event rates after 6 months; had significantly lower hospital costs ($9,658 +/- 5,287 vs. $11,604 +/- 6,125) and were discharged 3 days earlier |
| **Use of discounting** | None stated | None stated | None stated |

| **Primary author** | | **Rodriguez, A.** | **Rossouw, J. E.** | **Oyewo, E. A.** |
| --- | --- | --- | --- | --- |
| **Pub Year** | | 1993 | 1993 | 1989 |
| **Title** | | Argentine randomized trial of percutaneous transluminal coronary angioplasty versus coronary artery bypass surgery in multivessel disease (ERACI): in-hospital results and 1-year follow-up. ERACI Group | Community-based intervention: the Coronary Risk Factor Study (CORIS) | A therapeutic audit in the management of hypertension in Nigerians |
| **Journal** | | J.Am.Coll.Cardiol. | Int.J.Epidemiol. | East Afr.Med.J. |
| **Type of study** | | CCA | CCA | CEA-CL |
| **Study Setting** | | Single Nation :  Argentina | Single Nation:  South Africa | Single Nation:  Nigeria |
| **Size of a) intervention and b) control group in study** | | a) PTCA-Stent: n=63  b) By-pass grafting: n=64 | *Population at baseline (and at follow-up)*  a) High intensity intervention: n=2278  (n=2040); Cohort for study n=1251  b) Low intensity intervention: n=2620  (n=2494); Cohort for study n=1531  Control Group: n=2290 (n=2259); Cohort  for study n=1305 | 1. Diurelics    1. Thiazides: n=267    2. Frusemide: n=100 2. Central sympathoplegic and diuretics    1. Thiazides + Methyldopa: n=272 3. Central sympathoplegic with diuretics and vasodilator    1. Thiazides+MethyldoPa + Hydralazine: n=62 4. Vasodílator alone    1. Hydralazine: n=18 5. Betablockers and diuretics    1. Propranol+Thiazides: n=58 6. Fixed drug combinalions    1. "Brinerdine": n=69    2. "Minizide": n=39 |
| **Data generation** | | RCT | Observational Trial | Other: Cross-sectional |
|  | **Intervention Target** | Revascularization | Risk factor:  Multiple | Risk factor:  High Blood Pressure |
|  | **Type of Intervention** | Case Management:  Treatment | Primary Prevention:  Population Based | Various:  Personal Intervention |
|  | **Type of measure** | Procedure | Social marketing | Pharmaceutical |
|  | **Benefit measure** | Avoided CVD incidents | Behavior Change | Blood Pressure |
|  | **Funding source** | Foundation | Not stated | Not stated |
|  | **Economic perspective** | Health Care Sector | Not stated | Not stated |
|  | **Study length and follow-up /**  **time period applied in model** | 1 year; 3 years; and 5 years follow-up after intervention | 4 years of intervention; no further follow-up reported | Cross-sectional observation, no follow-up reported |
|  | **Target Group:**  **1. Risk factor/Co-morbidity**  **2. Male/Female ratio**  **3. Age**  **4. Socioeconomic Status**  **5. Other underlying factors** | 1. Multivessel coronary artery disease 2. Female/Male:   CABG (11/89)  PTCA (19/81)   1. CABG: 55 +/- 8   PTCA: 59 +/- 2   1. Not stated | 1. Total Cholesterol, High Blood Pressure, Presence of Smoking, Elevated BMI 2. Control: Female/Male (54.4/45.6); LII: Female/Male (53.6/46.4) HII: F/M(56.4/43.6) 3. 15-64 years 4. Largely middle class 5. White populations of three rural towns | 1. Hypertension 2. Female/Male (25/75) 3. 25-44 4. Not stated 5. All from same outpatient clinic; drugs were already assigned at the time of evaluation |
|  | **Intervention (detail)** | Transluminal coronary angioplasty (PTCA) | LII: Report of baseline results to participants, health education programme, and mass media programme (six different billboards,  six posters, eight mailings, frequent news items, health messages on electricity accounts, and one special supplementin a local newspaper); HII: Same as LII plus additional info on risk factor management, public meetings by experts on each risk factor, liaison with community organizations, establishment of community committees to co-ordinate and encourage health activities, participation in generating and testing of educational materials, interpersonal small-group intervention to high-risk individuals, and active follow-up of hypertensives as opposed to the passive availability of a blood pressure station | Efficacy of different anti-hypertensive drugs, the patients were already assigned to. Including:  a)Thiazide (50mg);  b)Methyldopa (750mg);  c)Hydralazine (50mg);  d)Thiazide + Methyìdopa;  e)Thiazide +. Methyldopa + Hydralazine;  f)Propranolol (80mg)  g)Propranolol + Thiazide  h)"Brinerdine"  i)"Minizide" |
|  | **Comparator (detail)** | Coronary artery bypass graft surgery (CABG) | No intervention | Respective alternative drug |
|  | **Results (detail)** | At 1-year-follow-up, patients treated with coronary artery bypass grafting were more frequently free of angina, reinterventions and  combined cardiac events than were patients treated with coronary angioplasty (83.5% vs. 63,7%p < 0.005).  Cumulative (group) costs at 1-year: PTCA ($438,000), CABG ($828,000) | $5 per capita cost for LII; $22 per capita cost for HII; but very similar improvement rates for both groups (difference was insignificant) | (Month.Costs/Mean Effec../EC-ratio)  a) 6.00/ 2.94/ 0.49  d)15.00/ 4.05/ 0.27  e) 27/ 4.95/ 0.18  f) 12.00/ 3.1/ 0.26  g) 18.00/ 2.53/ 0.14  h) 15.00/ 3.2/ 0.21  i) 21.00/ 1.3/ 0.06  (Efficacy by Score: 1=BP no change or higher - 5=BP <140/90) |
|  | **Use of discounting** | None stated | None stated | None stated |

**Table of detailed results from included studies 2/2 (Modeling studies) – *please note, price levels are not standardized***

| **Primary author** | **Ribeiro, RA** | **Rubinstein, A** | **Ker, J. A.** |
| --- | --- | --- | --- |
| **Pub Year** | 2009 | 2009 | 2008 |
| **Title** | Cost-Effectiveness of implantable cardioverter-defibrillators in Brazil: Primary Prevention Analysis in the Public Sector | Generalized cost-effectiveness analysis of a package of interventions  to reduce cardiovascular disease in Buenos Aires, Argentina | Decision-making using absolute cardiovascular risk reduction and incremental cost-effectiveness ratios: a case study |
| **Journal** | Value in Health | Cost Effectiveness and Resource Allocation | Cardiovasc.J.Afr. |
| **Type of study** | CUA | CUA | CEA-CL |
| **Study Setting** | Single Nation:  Brazil | Single Nation:  Argentina | Single Nation:  South Africa |
| **Data generation** | Modeling | Modeling | Modeling |
| **Type and source of effectiveness data** | Metaanalysis of clinical trials (Developed countries) | Metaanalysis of clinical trials (Developed countries) | Metaanalysis of clinical trials (Developed countries) |
| **Intervention Target** | Other: Fatal arrhythmia | Risk factor:  Various | Risk factor:  Multiple |
| **Type of Intervention** | Secondary Prevention | Primary Prevention:  Personal & Population | Primary Prevention:  Personal Intervention |
| **Type of measure** | Medical Technology | Various | Pharmaceutical |
| **Benefit measure** | QALYs | DALYs | Other |
| **Funding source** | Government | Government | Industry |
| **Economic perspective** | Health Care Sector | Health Insurance (TPP) | Not stated |
| **Time period applied in model** | 20 years | 10 years | No time period stated, hypothetical comparison of status quo |
| **Target Group:**  **1. Risk factor/Co-morbidity**  **2. Male/Female ratio**  **3. Age**  **4. Socioeconomic Status**  **5. Other underlying factors** | 1. Congestive Heart Failure (CHF) NYHA II and III  2. Not stated  3. 60 years  4. Not stated | 1. Stratified by CVD risk 2. Not specified 3. Not stated 4. Uninsured | 1. Male smoker, BP 160/100mmHg, total cholesterol 6 mmol/l, low-density lipoprotein (LDL) cholesterol 4.2 mmol/l, and high-density lipoprotein (HDL) cholesterol 0.7 mmol/l 2. Male 3. 56 4. not stated 5. Single Person Case Study |
| **Intervention (detail)** | ICD implant in a hypothetical cohort of CHF patients with NYHA II and III without prior event of life-threatening arrhythmia | Six individual based interventions delivered to the uninsured population in  Buenos Aires (26.5% of total population): treatment of hypertension through lifestyle change and drugs;  treatment of hypercholesterolaemia through a low-cholesterol diet and statins;  smoking cessation by drug therapy with bupropion for two months;  modified polypill strategy (thiazides 25mg, enalapril 10mg, atorvastatin 10mg, and aspirin 100mg) for individuals with an estimated combined risk of cardiovascular events over the next decade above 5%; modified polypill strategy for those with a risk above 10%; and modified polypill strategy for those with a risk above 20%.  Two population-based interventions: cooperation between government, consumer association, and bakery chambers to  reduce salt in bread (a reduction of 1g of salt per 100g of bread); and mass education strategies to reduce hypertension,hypercholesterolaemia, and obesity. | Quit smoking;  a) Hypertension treatment with perindopril-indapamide  combination (4 mg/2.5 mg);b) Lipid treatment Atorvastatin 10 mg;  c) Lipid treatment Atorvastatin 40mg  d) Hypertension treatment and lipid treatment Atorvastatin 10mg |
| **Comparator (detail)** | Conventional treatment not further specified | No intervention | No treatment |
| **Results (detail)** | Conventional treatment:  Total cost (US$ PPP): 24,619  Mean life years: 5.95 Mean QALYs: 5.23  ICD therapy:  Total cost (US$ PPP): 70,841  Mean life years: 6.99  Mean QALYs: 6.15  ICER:  (US$PPP/LY): 44,304  (US$PPP/QALY): 50,345 | The total costs were ARS 87,471 with less salt in bread; ARS 634,069 with mass media campaign; ARS  23,533,467 with polypill for over 20% CVD risk; ARS 46,323,335 with polypill for over 10% CVD risk; ARS 63,893,600 with polypill for over 5% CVD risk; ARS 37,478,853 with blood pressure lowering therapy; ARS 12,317,628 with smoking cessation therapy;  and ARS 40,253,626 with treatment for hypercolesterolaemia.  The DALYs were 579 with less salt in bread; 1,158 with mass media campaign; 6,539 with polypill for over 20% CVD risk; 11,263 with polypill for over 10% CVD risk; 14,095 with polypill for over 5% CVD risk; 4,857 with blood pressure lowering therapy; 367 with smoking cessation therapy; and 567 with treatment of hypercolesterolaemia.  The average cost-utility ratios (or incremental costs per DALY over no intervention) were ARS 151 with less salt; ARS 547 with  mass media; ARS 3,599 with polypill over 20%; ARS 4,113 with polypill over 10%; ARS 4,533 with polypill over 5%; ARS 7,716 with blood pressure therapy; ARS 33,563 with smoking cessation; and ARS 70,994 with hypercholesterolaemia treatment.. | Costs per % of risk reduction:   1. R21.35 2. R22.93 3. R12.77 4. R23.84 |
| **Use of discounting** | Costs/Benefits at 3% p.a. | Costs/Benefits at 3% p.a | None stated |

| **Primary author** | **Araujo, D. V.** | **Thavorn, K.** | **Lim, S. S.** |
| --- | --- | --- | --- |
| **Pub Year** | 2008 | 2008 | 2007 |
| **Title** | Cost-effectiveness of prehospital versus inhospital thrombolysis in acute myocardial infarction | A cost-effectiveness analysis of a community pharmacist-based smoking cessation programme in Thailand | Prevention of cardiovascular disease in high-risk individuals in low-income and middle-income countries: health effects and costs |
| **Journal** | Arq Bras.Cardiol. | Tob.Control | Lancet |
| **Type of study** | CEA-LY | CEA-LY | CCA |
| **Study Setting** | Single Nation:  Brazil | Single Nation:  Thailand | Multi-National:  Cross Country |
| **Data generation** | Modeling | Modeling | Modeling |
| **Type and source of effectiveness data** | RCT (Developed Country) | Metaanalysis of clinical studies (Developed countries) | Metaanalysis of clinical studies (Developed countries) |
| **Intervention Target** | Revascularization | Risk factor:  Smoking | Various |
| **Type of Intervention** | Case Management:  Treatment | Primary Prevention:  Personal Intervention | Secondary Prevention:  Personal Intervention |
| **Type of measure** | Procedure | Health Education | Pharmaceutical |
| **Benefit measure** | Life Years | Life Years | CVD related incidence |
| **Funding source** | Other | Government | Not stated |
| **Economic perspective** | Health Insurance | Health Care Sector | Not stated |
| **Time period applied in model** | 20 years | Lifetime | 10 years |
| **Target Group:**  **1. Risk factor/Co-morbidity**  **2. Male/Female ratio**  **3. Age**  **4. Socioeconomic Status**  **5. Other underlying factors** | 1. ST-elevation myocardial infarction (STEMI) 2. Not Stated 3. Not Stated 4. Not Stated | 1. Smoking (10-20 cigarrettes/day) 2. Female/Male (100/100) 3. 40, 50 and 60 years 4. Not Stated 5. Simulated cohorts | 1. High risk for CVD 2. Not Stated 3. 40-79 4. low and middle income countries |
| **Intervention (detail)** | Prehospital Thrombolysis | CPSC programme: systematic identification and documentation of smoking status; provision of a personalised and supportive advice on smoking cessation; an assessment a smoker’s interest in quitting and level of nicotine dependence; and the provision of appropriate therapy with self-help materials and seven scheduled 10-minute follow-up visits | Individuals w/ existing CVD would receive aspirin, an angiotensin-converting-enzyme inhibitor, a β blocker, and a statin. Individuals w/o existing CVD but who are at high risk would receive aspirin, an angiotensin-converting-enzyme inhibitor, a thiazide, and a statin |
| **Comparator (detail)** | Inhospital Thrombolysis | Usual care: discussion on smoking status, assessment of motivation and nicotine dependence, provision of brief advice and support as well as provision of therapy without follow-up care | No treatment |
| **Results (detail)** | Incremental cost effectiveness ratio of R$176 per .1585 life year gained (over 20 years) with Prehospital thrombolysis | Age 40 cohort: Cost savings of 17 503.53 baht (£250; J325; $500) to the health system and life year gains of 0.18 years for men; costs savings of 21 499.75 baht (£307; J399; $614) and life year gains of 0.24 years for women | The intervention could avert almost 18 million deaths in 23 low-income and middle-income countries over the next  10 years. The financial resources needed to scale-up this intervention are an average investment per year of around $5 billion, or $1.08 per head. |
| **Use of discounting** | None stated | 3% for both costs and outcomes | None stated |

| **Primary author** | **Polanczyk, C. A.** | **Araujo, DV** | **Robberstad, B** |
| --- | --- | --- | --- |
| **Pub Year** | 2007 | 2007 | 2007 |
| **Title** | Cost-effectiveness of sirolimus-eluting stents in percutaneous coronary interventions in Brazil | Cost-effectiveness and budget impact analysis of rosuvastatin and atorvastatin for LDL-cholesterol and cardiovascular events lowering within the SUS* scenario | Cost-effectiveness of medical interventions to prevent  cardiovascular disease in a sub-Saharan African country – the case of Tanzania |
| **Journal** | Arq Bras.Cardiol. | Int J Atheroscler | Cost Effectiveness and Resource Allocation. |
| **Type of study** | CEA-LY | CEA - LY | CUA |
| **Study Setting** | Single Nation:  Brazil | Single Nation:  Brazil | Single Nation :  Tanzania |
| **Data generation** | Modeling | Modeling | Modeling |
| **Type and source of effectiveness data** | Metaanalysis of clinical studies (Developed countries) | Metaanalysis of clinical studies (Developed countries) | Metaanalysis of clinical studies (Developed countries) |
| **Intervention Target** | Revascularization | Risk factor:  Dyslipidemia | Risk factor:  Various |
| **Type of Intervention** | Case Management:  Treatment | Primary Prevention:  Personal Intervention | Primary Prevention:  Personal Intervention |
| **Type of measure** | Medical Technology | Pharmaceutical | Pharmaceutical |
| **Benefit measure** | Life Years | Life years gained | DALYs |
| **Funding source** | Industry | Not stated | Government |
| **Economic perspective** | Health Insurance | Health Insurance | Not stated |
| **Time period applied in model** | Lifetime | 20 years | Lifetime |
| **Target Group:**  **1. Risk factor/Co-morbidity**  **2. Male/Female ratio**  **3. Age**  **4. Socioeconomic Status**  **5. Other underlying factors** | 1. Coronary Lesions 2. Not Stated 3. 50 years 4. Not Stated | 1. Dyslipidemia, Hypertension, Diabetes 2. Not specified 3. Not stated 4. Not stated | 1. CVD risk defined as low, medium, high 2. Not specified 3. >45 4. Not stated |
| **Intervention (detail)** | PCI with sirolimus-eluting stents, and PCI with BMS followed by SES for symptomatic restenosis | Treatment with the statin Rosuvastatin | fourteen medical interventions of primary prevention of  cardiovascular disease in Tanzania, including Acetylsalicylic acid, a diuretic drug (Hydrochlorothiazide), a ß-blocker (Atenolol), a calcium channel blocker (Nifedepine), a statin (Lovastatin) and various  combinations of these, including a hypothetical polypill |
| **Comparator (detail)** | Percutaneous coronary intervention (PCI) with bare-metal stent | Treatment with the statin Atorvastatin | No intervention |
| **Results (detail)** | SES had an incremental cost-effectiveness ratio of R$27,403 per event avoided in one year for non-public institutions, incremental cost-effectiveness ratio of R$47,529 per event avoided in one year for public institutions | Costs:  R: R$8350.08  A: R$8612.52  Benefits  (Life years gained):  R: 9.32  A:9.26  C/E-ratio:  Atorvastatin is dominant to Rosuvastatin | C/E ratio ranged from 85 USD per DALY for Hydrochlorothiazide to 4589 USD for a polypill in low risk  patients |
| **Use of discounting** | 3% per year | Costs/Benefits at 7% p.a. | Standardized discount rate  from 10% to 3% |

| **Primary author** | **Wessels, F.** | **Asaria, P.** | **Gaziano, T. A.** |
| --- | --- | --- | --- |
| **Pub Year** | 2007 | 2007 | 2006 |
| **Title** | Eprosartan in secondary prevention of stroke: the economic evidence | Chronic disease prevention: health effects and financial costs of strategies to reduce salt intake and control tobacco use | Cardiovascular disease prevention with a multidrug regimen in the developing world: a cost-effectiveness analysis |
| **Journal** | Cardiovasc.J.Afr. | Lancet | Lancet |
| **Type of study** | CUA | CCA | CUA |
| **Study Setting** | Single Nation:  South Africa | Multi-National:  Cross Country - By income level stratified | Multi-National:  Worldbank regions |
| **Data generation** | Modeling | Modeling | Modeling |
| **Type and source of effectiveness data** | RCT (Developed Country) | Metaanalysis of clinical studies (Developed countries) | Metaanalysis of clinical studies (Developed countries) |
| **Intervention Target** | Risk factor:  High Blood Pressure | Risk factor:  Multiple | Risk factor:  Multiple |
| **Type of Intervention** | Secondary Prevention | Primary Prevention:  Population Based | Various:  Personal Intervention |
| **Type of measure** | Pharmaceutical | Various | Pharmaceutical |
| **Benefit measure** | QALYs | Avoided CVD incidents | QALYs |
| **Funding source** | Not stated | Not stated | Government |
| **Economic perspective** | Health Insurance | Not stated | Not stated |
| **Time period applied in model** | 2.5 years treatment; lifetime follow-up | 12 months | Lifetime |
| **Target Group:**  **1. Risk factor/Co-morbidity**  **2. Male/Female ratio**  **3. Age**  **4. Socioeconomic Status**  **5. Other underlying factors** | 1. Stroke 2. Not Stated 3. Not Stated 4. Not Stated | 1. Smoking and High Blood Pressure 2. Not Stated 3. 30-100 years 4. 23 low and middle income countries | 1. High Blood Pressure 2. Not Stated 3. 35–74 years 4. The six low-income and middle-income regions defined by the World Bank |
| **Intervention (detail)** | Eprosartan for Secondary Prevention | Allowing AF to persist and develop into permanent arrhythmia with controlled ventricular rate | 1. Salt reduction strategy 2. Tobacco Control through taxation, comprehensive bans on smoking in the workplace, mass media campaigns, bans on advertising |
| **Comparator (detail)** | Use of Amlodipine and Perindopril | Restoration and maintenance of sinus rhythm (SR) through mainly antiarrhythmic therapy | No treatment |
| **Results (detail)** | A cost−utility analysis of eprosartan estimated a cost saving of ZAR 53 132 per quality-adjusted life year gained compared with amlodipine, and a cost saving of ZAR 72 888 compared with perindopril | Cost/patient of pharmacological ventricular rate control = €1225.00; Cost/patient of cardioversion and subsequent SR maintenance = €2526.00 | Overall CVD deaths averted over 10 years: 10.45 million  Costs: Less than US$0.40 per person per year in low-income and lower middle-income countries, and US$0.50–1.00 per person per year in upper middle-income countries (as  of 2005) |
| **Use of discounting** | 5% | Not discounted due to 12 month study time | None stated |

| **Primary author** | **Biccard, B. M.** | **Gaziano, T. A.** | **Gaziano, T. A.** |
| --- | --- | --- | --- |
| **Pub Year** | 2006 | 2005 | 2005 |
| **Title** | The pharmaco-economics of peri-operative beta-blocker and statin therapy in South Africa | Cost-effectiveness analysis of hypertension guidelines in South Africa: absolute risk versus blood pressure level | Cardiovascular disease in the developing world and its cost-effective management |
| **Journal** | S.Afr.Med.J. | Circulation | Circulation |
| **Type of study** | CCA | CUA | CUA |
| **Study Setting** | Single Nation:  South Africa | Single Nation:  South Africa | Multi-National:  Worldbank regions |
| **Data generation** | Modeling | Modeling | Modeling |
| **Type and source of effectiveness data** | RCT (Developed Country) | Metaanalysis of clinical studies (Developed countries) | Metaanalysis of clinical studies (Developed countries) |
| **Intervention Target** | Risk factor:  Multiple | Risk factor:  Multiple | Various |
| **Type of Intervention** | Secondary Prevention | Primary Prevention:  Personal Intervention | Multiple |
| **Type of measure** | Pharmaceutical | Pharmaceutical | Various |
| **Benefit measure** | Avoided CVD incidents | QALYs | QALYs |
| **Funding source** | Not stated | Not stated | Not stated |
| **Economic perspective** | Health Insurance | Health Insurance | Not stated |
| **Time period applied in model** | 30 days | Lifetime | Lifetime |
| **Target Group:**  **1. Risk factor/Co-morbidity**  **2. Male/Female ratio**  **3. Age**  **4. Socioeconomic Status**  **5. Other underlying factors** | 1. Elective surgery with an expected peri-operative major cardiovascular complication rate exceeding 10% 2. Not Stated 3. Mean age of 64 years 4. Not Stated | 1. High Blood Pressure and Absolute CVD Risk 2. Not Stated 3. 35-75 years 4. Not stated | 1. Multiple: Acute MI, Secondary Prevention, CHF 2. Not stated 3. Not Stated 4. Not Stated |
| **Intervention (detail)** | Pateints had already been prescribed an antihypertensive: calcium channel blocker (CCB) (129/455; 28.4%), FDC co-amiloride (95; 20.9%), angiotensin-converting enzyme inhibitor (ACEI) (70; 15.4%), a-methyldopa (a-MD) (70; 15.4%), b-blockers (65; 14.3%), FDC reserpineþclopamideþdihydro- ergocristine (13; 2.9%), FDC atenololþchlorthalidone (9; 2%) and prazosin, and a-blocker (4; 0.9%) | Drug treatment initiation at 2 different blood pressure levels (160/95 and 140/90 mm Hg) and 4 different levels of absolute CVD risk over 10 years (40%, 30%, 20%, and 15%) | Multiple: Acute MI = 4 Strategies (aspirin; aspirin and atenolol; aspirin, atenolol, and streptokinase; and aspirin, atenolol, and tissue plasminogen activator (tPA)), Secondary Prevention (aspirin at 75 to 100 mg/d, atenolol at 100 mg/d, enalapril at 10 mg/d, and lovastatin at 40 mg/d), CHF (addition of an ACE inhibitor (enalapril and/or metoprolol)to a baseline of diuretic treatment |
| **Comparator (detail)** | Respective drug | No Treatment | No Treatment |
| **Results (detail)** | CER and relative CER for each drug combination - most cost-effective was co-amiloride with CER 42.9, least was combination was CCB with ACEI, CER 3145.2 | incremental cost-effectiveness ratios for treating those with 10-year absolute risk for CVD 40%, 30%, 20%, and 15% were $700, $1600, $4900, and $11 000 per quality-adjusted life-year gained, respectively | Multiple: Acute MI (Incremental cost per QALY gained for both aspirin and ß-blocker < $25 for all 6 regions; streptokinase $630 < $730 ; ICERs for tPA = approx. $16 000 compared w/ streptokinase), Secondary Prevent: w/ hospitals (ICERs per QALY gained: combination of aspirin, atenolol, and enalapril = $660 sub-Saharan Africa region < $866 Europe and Central Asia region. All 4 medications = $1700<$2000 per QALY gained all regions), w/o hospitals (ICERs per QALY: aspirin and ß-blockers = $386 South Asia region < $545 Latin America and the Caribbean region. + enalapril = $783 sub-Saharan Africa < $1111 Latin America and the  Caribbean. + lovastatin = $2000 < $2500 over all regions), CHD: w/ hospitals (enalapril = cost-saving, ICER for metoprolol = $120 < $220 per QALYgained); w/o hospitals (ACEI = $30 per QALY gained,B-blocker ICER =approximately $275 |
| **Use of discounting** | None stated | 3% per year | None stated |

| **Primary author** | **Murray, C. J.** | **Anderson, A. N.** | **Hauswald, M.** |
| --- | --- | --- | --- |
| **Pub Year** | 2003 | 2000 | 1997 |
| **Title** | Effectiveness and costs of interventions to lower systolic blood pressure and cholesterol: a global and regional analysis on reduction of cardiovascular-disease risk | AT1 receptor blockers--cost-effectiveness within the South African context | Designing a prehospital system for a developing country: estimated cost and benefits |
| **Journal** | Lancet | S.Afr.Med.J. | Am.J.Emerg.Med. |
| **Type of study** | CUA | CEA-CL | CCA |
| **Study Setting** | Multi-National:  WHO regions | Single Nation:  South Africa | Single Nation:  Malaysia |
| **Data generation** | Modeling | Modeling | Modeling |
| **Type and source of effectiveness data** | Metaanalysis of clinical studies (Developed countries) | Metaanalysis of clinical studies (Developed countries) | Observational trial (Developed country) |
| **Intervention Target** | Risk factor:  Multiple | Risk factor:  High Blood Pressure | Resuscitation/ Revascularization |
| **Type of Intervention** | Primary Prevention:  Population based and Personal intervention | Various:  Personal Intervention | Case Management:  Treatment |
| **Type of measure** | Various | Pharmaceutical | Procedure |
| **Benefit measure** | DALYs | Blood Pressure | Avoided CVD incidents |
| **Funding source** | Not stated | Industry | Not stated |
| **Economic perspective** | Not stated | Health Insurance | Not stated |
| **Time period applied in model** | Lifetime | 12 months | 1 year |
| **Target Group:**  **1. Risk factor/Co-morbidity**  **2. Male/Female ratio**  **3. Age**  **4. Socioeconomic Status**  **5. Other underlying factors** | 1. Absolute CVD Risk and multiple risk factors 2. Varied by subregion 3. Varied by subregion 4. Varied by subregion 5. 14 epidemiological subregions of the world identified by the WHO CHOICE program | 1. Mild to moderate hypertension 2. Not Stated 3. Not Stated 4. Not Stated | 1. Post MI 2. Not stated 3. Not stated 4. Not stated |
| **Intervention (detail)** | 17 non-personal and personal health-service interventions and combinations thereof | AT1 receptor blockers (ARBs) candesartan 16 mg (Atacand), losartan 50 mg (Cozaar), valsartan 80 mg (Diovan) and irbesartan 150 mg (Aprovel) | Introduction of an emergency medical services (EMS) system according to US standards in the City of Kuala Lumpur, to save more citizens after Myocardial Infarction |
| **Comparator (detail)** | Alternative strategy | Alternative drug | Current Care (performed by police / private vehicle) |
| **Results (detail)** | In all regions, the four non-personal interventions (Very Cost effective) have cost-effectiveness ratios that are lower than personal health-service interventions; Treatment of SBP> 160 mm Hg is very cost effective in all regions. Treatment of people w/ total cholesterol concentrations > 6·2 mmol/L using statins is very cost effective in all regions; Treatment of people w/ absolute risk >35% is the most cost effective personal intervention | Two measures: reduction in SDBP per R100 spen = Candesartan was most cost-effectiveat 4.48 mmHg/R1OO. Losartan was 3.77, Irbesartan was 3.37 and and Valsartan was 3.04 mmHg/R100. Cost to achieve 1mmHG reduction in SDBP = candesartan R22.34/mmHg, losartan (R26.54/ mmHg), irbesartan (R29.65/ mrnHg) and valsartan (R32.86/ mrnHg) | A prehospital system for Kuala Lumpur would cost approximately $2.5 million per year.  It might save seven lives, three of which would be marred by significant neurological injury. |
| **Use of discounting** | All costs at 3% | None stated | None stated |
